# Reducing demographic bias in biomedical machine learning for cancer detection using cfDNA methylation

**DOI:** 10.1186/s13059-026-04006-0

**Published:** 2026-02-25

**Authors:** Shuo Li, Weihua Zeng, Wenyuan Li, Chun-Chi Liu, Yonggang Zhou, Xiaohui Ni, Mary L. Stackpole, Angela H. Yeh, Andrew Melehy, David S. Lu, Steven S. Raman, William Hsu, Lopa Mishra, Kirti Shetty, Benjamin Tran, Megumi Yokomizo, Preeti Ahuja, Yazhen Zhu, Hsian-Rong Tseng, Denise R. Aberle, Vatche G. Agopian, Steven-Huy B. Han, Samuel W. French, Steven M. Dubinett, Xianghong Jasmine Zhou, Wing Hung Wong

**Affiliations:** 1 Department of Pathology and Laboratory Medicine, David Geffen School of Medicine, University of California at Los Angeles, Los Angeles, CA 90095 USA; 2 EarlyDiagnostics Inc., Los Angeles, CA 90095 USA; 3 Department of Surgery, David Geffen School of Medicine, University of California at Los Angeles, Los Angeles, CA 90095 USA; 4 Department of Radiological Sciences, David Geffen School of Medicine, University of California at Los Angeles, Los Angeles, CA 90095 USA; 5 Jonsson Comprehensive Cancer Center, University of California at Los Angeles, Los Angeles, CA 90095 USA; 6 Institute for Bioelectronic Medicine, Divisions of Gastroenterology and Hepatology, Department of Medicine, Feinstein Institutes for Medical Research, Northwell Health, Manhasset, NY 11030 USA; 7 Department of Surgery, George Washington University, Washington, DC 20037 USA; 8 Department of Gastroenterology and Hepatology, the University of Maryland, School of Medicine, Baltimore, MD 21201 USA; 9 Department of Molecular and Medical Pharmacology, David Geffen School of Medicine, University of California at Los Angeles, Los Angeles, CA 90095 USA; 10 Department of Medicine, David Geffen School of Medicine, University of California at Los Angeles, Los Angeles, CA 90095 USA; 11 VA Greater Los Angeles Health Care System, Los Angeles, CA 90073 USA; 12 Institute for Quantitative and Computational Biosciences, University of California at Los Angeles, Los Angeles, CA 90095 USA; 13 Department of Statistics, Stanford University, Stanford, CA 94305 USA; 14 Department of Biomedical Data Science, Stanford University, Stanford, CA 94305 USA

**Keywords:** Demographic bias, cfDNA methylation, Bias correction, Machine learning, Cancer detection

## Abstract

**Background:**

Machine learning models in biomedical research are often hindered by demographic imbalances in clinical datasets, leading to biased predictions that disadvantage minority populations. Existing bias-correction methods face limitations in handling the heterogeneity of biomedical data and the complexity of demographic influences.

**Results:**

We present *DeBias*, a computational framework for mitigating demographic biases in high-dimensional biomedical datasets. *DeBias* identifies and removes bias-associated subspaces from the feature space using control samples, enabling global correction of demographic distortions while preserving disease-specific signals. To evaluate its effectiveness, we apply *DeBias* to cell-free DNA methylation data for cancer detection. *DeBias* achieves a significant reduction in the number of features exhibiting demographic bias and outperforms existing methods in improving cancer detection performance for minority populations. Performance gains are validated in independent cohorts, highlighting the robustness of the approach.

**Conclusions:**

*DeBias* offers an effective and generalizable strategy for correcting demographic biases in biomedical machine learning. It represents a step toward more equitable machine learning models that can deliver reliable and unbiased predictions across diverse patient populations.

**Supplementary Information:**

The online version contains supplementary material available at 10.1186/s13059-026-04006-0.

## Background

Machine learning and artificial intelligence have revolutionized biomedical research by enhancing the analysis of complex high-throughput data, such as genomic [1–3] and epigenomic [4–7] datasets. Despite their promise, a critical challenge arises from the demographic imbalances in training datasets, which often overrepresent the majority population due to different disease prevalence and systemic healthcare disparities [8, 9]. These imbalances compromise model performance in two key ways: (1) models are disproportionately optimized for majority population characteristics due to their overrepresentation in training data [10]; and (2) disparities in class distribution (e.g., case-to-control ratios across demographic groups) can confound demographic-specific patterns and disease-associated signatures. As a result, machine learning models trained on such data tend to inherit these imbalances, resulting in optimized performance for the majority group at the expense of accuracy for minority populations [8, 10]. These biases can impair predictive performance [10, 11], potentially causing serious or even life-threatening consequences for underrepresented patients. Consequently, addressing these imbalances in the training datasets is a fundamental challenge to ensure that machine learning models are fair, effective, and accurate in real-world healthcare settings.

Several strategies have been proposed to mitigate demographic biases, but each has notable limitations: (1) The most effective solution would be to incorporate more samples from minority populations to balance datasets [12], but this is often infeasible due to limited healthcare access and the underrepresentation of minority groups in biomedical research. (2) An alternative approach is to develop models trained exclusively on data from minority populations [13], thereby eliminating any influence from the majority group. However, the limited availability of samples from minority populations compromises model reliability and generalizability. (3) Oversampling the minority population [14, 15] can help balance datasets, but it unavoidably amplifies inherent variations within small, unrepresentative cohorts, introducing artificial biases and leading to overcorrection. (4) Most existing bias correction methods focus on technical biases (e.g., batch effects) [16–19], yet they do not fully account for complex demographic biases driven by both genetic and environmental factors. (5) Another possible approach is to exclude biased features from the dataset [20]. However, genetic and environmental factors often exert global effects on high-throughput data, making it impractical to correct bias by simply adjusting or removing a few features. (6) Deep learning-based methods have emerged to correct bias through two main strategies: first, employing autoencoders (AE, e.g., variational autoencoders [21–23]) to disentangle latent representations and isolate demographic bias from the desired bias-invariant patterns; second, directly penalizing the correlation between demographic features and model predictions to encourage the model to be invariant to demographic bias (e.g., adversarial learning [24]). While promising, these models typically require large amounts of diverse data to achieve reliable and generalizable performance. However, such conditions are not always met in real-world clinical datasets. Given the complexity of demographic biases in machine learning models, a more comprehensive approach is needed.

To overcome these limitations, we introduce *DeBias*, a novel computational framework that removes demographic biases in high-dimensional biomedical data, enabling more representative modeling particularly tailored to underrepresented populations. Rather than relying on feature-wise adjustments [17–19], *DeBias* identifies and eliminates bias-associated subspaces within the entire feature space, enabling global bias correction across all features. These bias-associated subspaces are detected by comparing control samples from majority and minority populations, effectively isolating demographic influences while preserving disease-related signals. By removing bias-associated subspaces across the dataset, *DeBias* leverages the large sample size of the majority population to enhance model performance for minority groups, while minimizing demographic distortions in predictions.

We evaluated *DeBias* in a clinically critical context: cancer detection using cell-free DNA (cfDNA) methylation data, and compared its performance against existing methods (i.e. SMOTE [15], ComBat [17], AE, and adversarial learning [24]). Specifically, we assessed its ability to: (1) reduce racial bias for African Americans in multi-cancer detection among the general population, and (2) eliminate ethnicity-related bias for Hispanic patients in liver cancer detection among high-risk liver disease patients. In both tasks, *DeBias* significantly reduced biases in individual methylation features and outperformed existing methods in improving cancer detection for underrepresented populations. These results highlight its potential as an effective approach for developing more accurate, reliable, and equitable machine learning models. Moreover, the principles behind *DeBias* extend beyond cancer detection and DNA methylation analysis, offering a generalizable solution for mitigating demographic biases in high-throughput biomedical data and other classification tasks.

## Results

### Overview of the *DeBias* framework

**Fig. 1:**
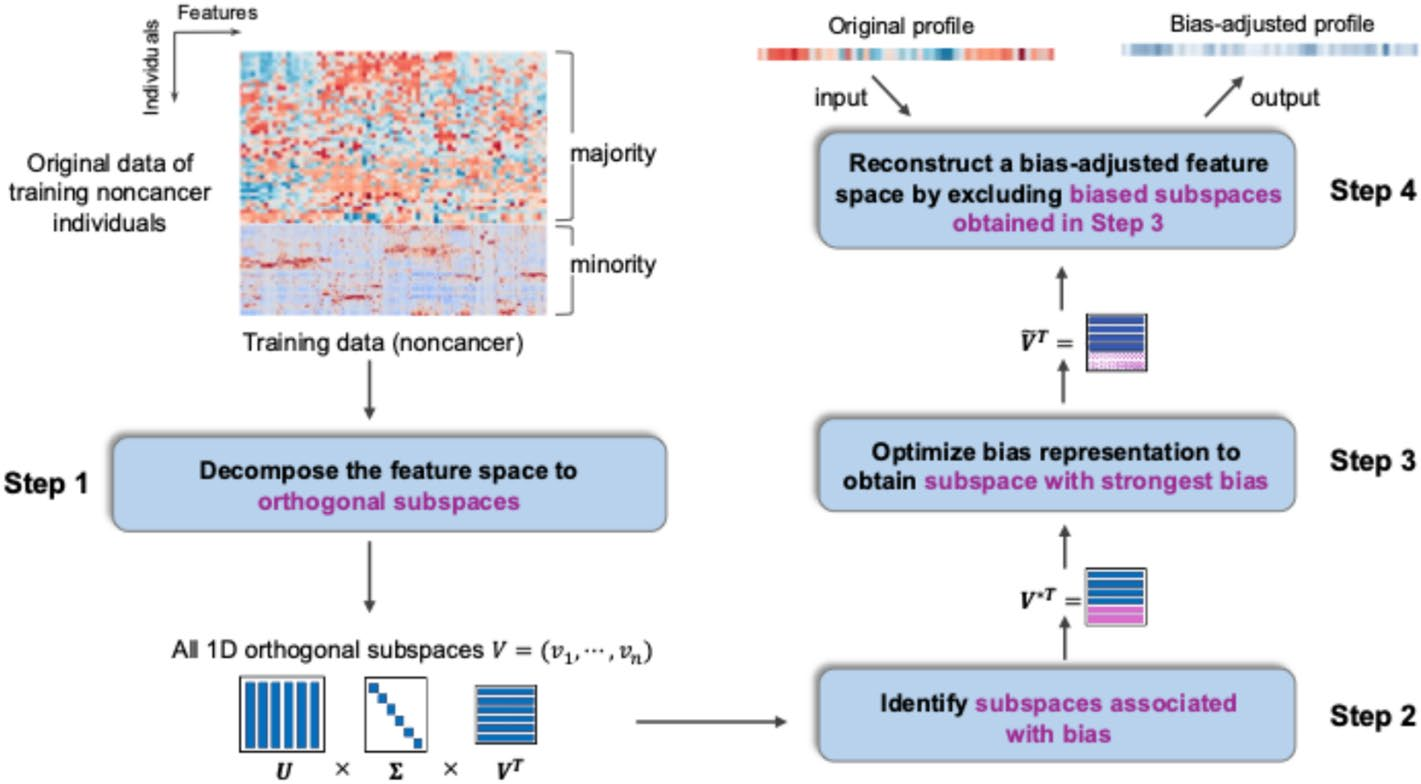
Overview of the *DeBias* pipeline for demographic bias correction in high-dimensional biomedical data. Given the feature matrix of a biomedical dataset as input, *DeBias* isolates and removes demographic bias while preserving disease-specific signals. It first applies PCA to control samples to decompose the feature space (Step 1), then isolates subspaces affected by bias through the dual criterion on bias- and disease-related effect sizes (Step 2). LDA refines these subspaces to maximize demographic separation (Step 3), and projections onto the biased components are removed to reconstruct a bias-adjusted feature space (Step 4). The output is a bias-adjusted matrix that enables unbiased downstream analyses for minority populations without compromising biological relevance

We developed a computational approach, *DeBias*, to address demographic biases in high-dimensional biomedical data by systematically isolating and removing bias-associated signals while preserving disease-specific signals. We assumed that the original dataset contains both disease and control samples, which was commonly satisfied in the classification and regression tasks, e.g. disease detection and monitoring. This approach operates through four steps (Fig. 1):

- Step 1: Decompose the feature space. To disentangle confounding demographic variation from disease-specific signals, we first decomposed the high-dimensional feature space into orthogonal, low-dimensional subspaces using principal component analysis (PCA) [25]. Critically, this decomposition was applied only to control samples from both majority and minority populations. By excluding disease samples, we ensured that subspaces primarily reflected demographic and non-disease-related biological variation. This step assumes that demographic effects manifest as linear components and that control samples can provide a sufficient representation of demographic structure. For $N$ control samples, we decomposed the biomedical feature matrix ${X}_{control}$ into ${X}_{control}=U\Sigma {V}^{T}$, where $V=({v}_{1},{v}_{2},\dots , {v}_{n})$ is the matrix of the principal components. Each column of $V$ naturally represented an orthogonal basis vector of the feature space, corresponding to an orthogonal 1-D subspace.
- Step 2: Identify subspaces linked to demographic biases. Next, we isolated subspaces exhibiting demographic biases toward minority populations without containing disease-specific signals. For each subspace $v$, we projected the training data matrix $X$ onto $v$ by calculating ${p}_{v}=Xv$ and computed two Cohen’s *d* effect sizes: one quantifying demographic bias between control samples from minority and majority populations, and another assessing disease-related differences between disease and control samples. Subspaces showing substantial demographic bias (effect size > ${es}_{bias}$, default ${es}_{bias}=0.1$) but minimal disease signals (effect size < ${es}_{disease}$, default ${es}_{disease}=0.5$) were flagged as biased subspaces, yielding two lists by the end of Step 2: biased subspaces $({v}_{1}^{\prime}, {v}_{2}^{\prime}, \dots , {v}_{m}^{\prime}$) and non-biased subspaces $({v}_{1}^{*}, {v}_{2}^{*}, \dots , {v}_{n-m}^{*}$). This design leverages the empirical observation that demographic and disease signals often occupy partially separable axes of variation in high-dimensional biomedical data, thereby enabling *DeBias* to selectively remove demographic distortions while preserving biologically meaningful disease signals.
- Step 3: Optimize bias representation via iterative linear discriminant analysis (LDA). While PCA subspaces capture dominant sources of variance, they may not optimally represent demographic bias, i.e. showing the strongest bias. To refine this, we applied LDA to the biased subspaces, iteratively constructing linear combinations ${(\widetilde{v}}_{(1)}, {\widetilde{v}}_{(2)}, \dots , {\widetilde{v}}_{(m)})$ that maximize demographic discrimination. Specifically, we created the most biased linear combination ${\widetilde{v}}_{(1)}$ of the biased subspaces $({v}_{1}^{\prime}, {v}_{2}^{\prime}, \dots , {v}_{m}^{\prime}$). We excluded the subspace spanned by ${\widetilde{v}}_{(1)}$ from the biased subspaces. On the remaining subspaces, we performed LDA to identify the next most biased linear combination ${\widetilde{v}}_{(2)}$ and iterated this process. Then we selected the linear combinations showing significant bias between minority and majority populations (effect size > ${es}_{bias}^{\prime}$, default ${es}_{bias}^{\prime}=0.1$), resulting in a final list of biased linear combinations ${V}_{bias}=({\widetilde{v}}_{\left(1\right)}, {\widetilde{v}}_{\left(2\right)}, \dots , {\widetilde{v}}_{\left(k\right)})$ and a final list of non-biased linear combinations ${V}_{nonbias}=({\widetilde{v}}_{\left(k+1\right)}, {\widetilde{v}}_{\left(k+2\right)}, \dots , {\widetilde{v}}_{\left(m\right)}) .$
- Step 4: Reconstruct a bias-adjusted feature space. Finally, we reconstructed the high-dimensional feature space by excluding projections onto the significantly biased linear combinations ${\mathrm{V}}_{{bias}}$ identified in Step 3. Formally, the reconstructed feature matrix was computed as ${X}_{reconstruct}=\mathrm{X}-{\sum }_{v\in {V}_{bias}}Xv{v}^{T}$, where $X$ is the original biomedical data matrix. The adjusted feature matrix retains the same dimensions as the original feature matrix. This operation globally removed demographic bias across the entire feature set while preserving disease-related variation. As a result, models trained on this bias-adjusted dataset leverage the statistical strength of the majority group without transferring demographic biases, improving generalizability and fairness across populations.

### Reducing racial bias in multi-cancer detection among African American patients

To evaluate the performance of *DeBias* in mitigating demographic biases, we applied it to a multi-cancer detection task using cfDNA methylation, where African American participants were underrepresented in the training set. Multi-cancer detection tests hold great promises in identifying multiple cancer types from a single noninvasive blood sample [4–7]; however, their accuracy in minority populations may be compromised due to the prevalent racial imbalances in clinical datasets (e.g., > 90% White participants in the PATHFINDER [26] and DETECT-A [27] trials). Correcting such bias is critical for ensuring the fairness and reliability of multi-cancer detection tests before clinical implementation.

**Fig. 2:**
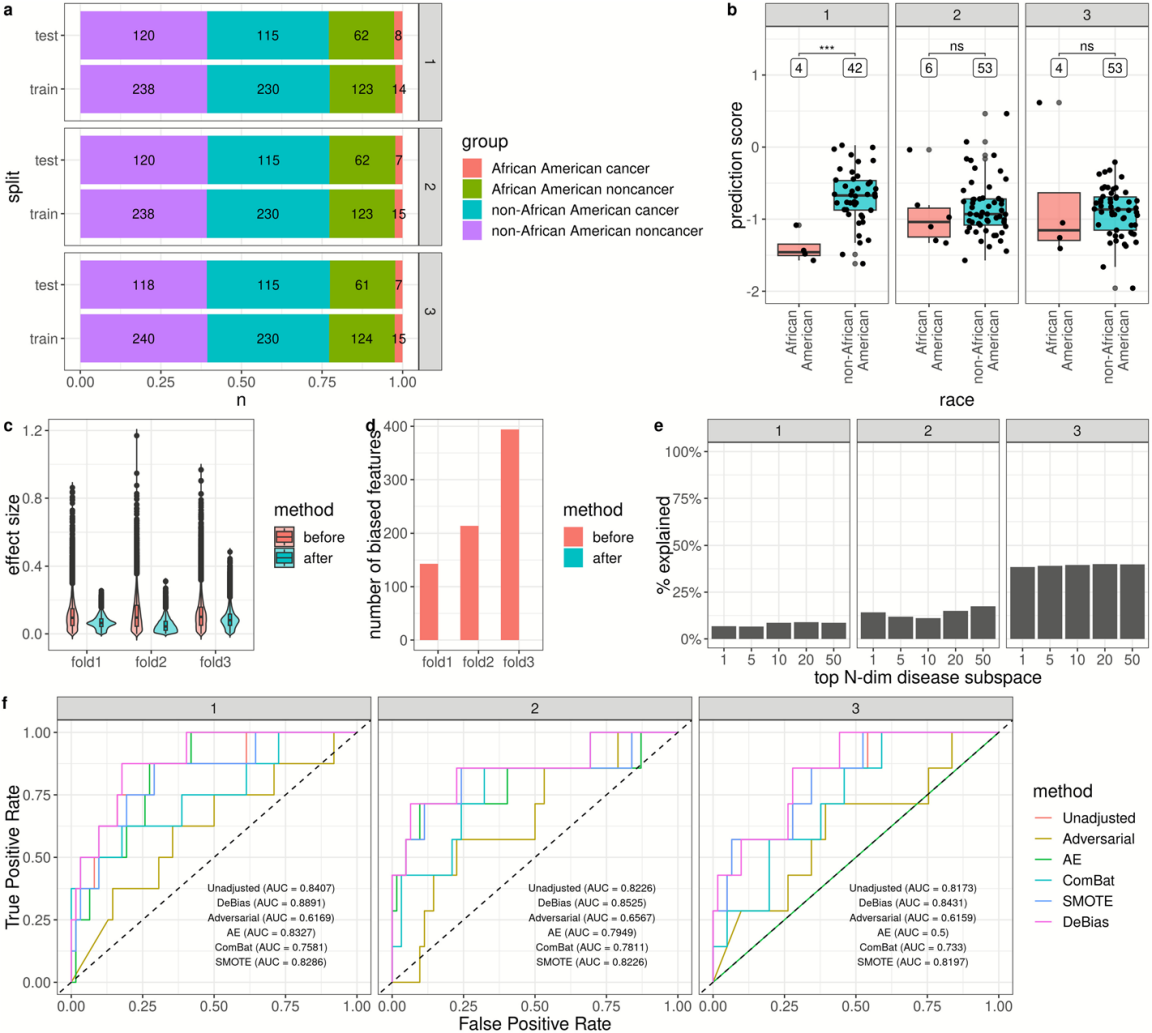
*DeBias* removed racial bias towards underrepresented African American populations in multi-cancer detection in three-fold cross-validation. **a** Composition of the training and testing datasets. **b** Distribution of cancer prediction scores for healthy African Americans and non-African Americans in the cross-validation testing set before bias correction. Statistical significance (*p*-value) was assessed using one-sided permutation t-test, comparing cancer prediction scores between African American and non-African American healthy samples. “***” denotes *p* < 0.001, and “ns” denotes *p* > = 0.05. **c** Distribution of race-associated effect size in individual features before and after *DeBias*. The race-associated effect size was evaluated between the African American and non-African American healthy samples. **d** Number of race-associated biased features before and after *DeBias*. **e** Proportion of variance in the top N-dimensional disease subspaces explained by the biased subspaces. **f** Receiver operating characteristic (ROC) curve for African American testing samples before bias correction (Unadjusted), after *DeBias* adjustment, and after alternative bias-correction methods. The facet labels in each subfigure indicated the testing fold ID

We analyzed a *MethylScan* plasma dataset comprising 703 non-African American samples (49.1% cancer) and 207 African American samples (10.6% cancer; Methods and Additional file 1: Table S1). *DeBias* was evaluated using three-fold cross-validation, with each fold preserving the original proportions of cancer versus noncancer samples and racial distributions (Fig. 2a). For classification, linear support-vector-machine (LSVM) models [4] were trained to distinguish between cancer and noncancer patients (see Methods). Because African American cancer cases were markedly underrepresented, the models tended to interpret race-associated methylation patterns in non-African American subjects as cancer signals. Consequently, healthy non-African American individuals exhibited higher cancer-like scores than healthy African Americans (one-sided permutation t-test *p*-value = 0.0004, 0.345, and 0.751 in Folds 1–3, respectively; Fig. 2b). The small number of healthy samples limited the statistical power to confirm these differences. Simultaneously, the area under the receiver operating characteristic curve (AUROC) before bias correction was lower for African Americans (AUC = 0.8407 with CI = [0.6892,0.9922], 0.8226 [0.5943,1.0000], 0.8173 [0.6534,0.9813] in Folds 1–3, and 0.8263 [0.7241,0.9285] overall) compared to non-African Americans (0.9475 [0.9174,0.9776], 0.9385 [0.9083,0.9687], 0.9634 [0.942,0.9849] in Folds 1–3, and 0.9485 [0.9328,0.9643] overall) and the combined cohort (0.9409 [0.9117,0.9702], 0.941 [0.9133,0.9686], and 0.9562 [0.9346,0.9777] in Folds 1–3, and 0.9446 [0.9294,0.9599] overall; Additional file 1: Table S2).

Next, we applied *DeBias* to identify race-associated biased subspaces (10, 11, and 12 dimensions for Folds 1–3, respectively) from the training folds and remove them from all folds (see Methods, Additional file 2: Figs. S1-S6). To measure the effectiveness of this bias reduction, we assessed differential methylation between healthy individuals of the two racial groups before and after bias correction, using the Cohen’s *d* effect size and permutation t-test. The race-associated effect sizes across individual features were significantly reduced after *DeBias* correction (permutation t-test *p*-value < 5e-05; Fig. 2c). Moreover, the number of race-associated differentially methylated features (Benjamini–Hochberg adjusted *p*-value < 0.05 from permutation t-tests) was substantially reduced after correction (Fig. 2d). Features flagged for racial bias before correction (0.9%, 1.4%, and 2.6% in Folds 1–3) dropped to 0.0% in all folds after correction. These results indicate that *DeBias* effectively removes demographic bias at the feature level through a global bias-removal procedure.

We next evaluated the impact of bias removal on cancer-associated signals. Cancer-specific subspaces were iteratively identified between the training cancer and noncancer cohorts using LDA (see Methods). We then quantified the proportion of variance in these cancer-specific subspaces explained by the biased subspaces identified through *DeBias*. On average, the biased subspaces accounted for 7.9%, 13.9%, and 39.4% of the variance (measured by the adjusted ${R}^{2}$ in the redundancy analysis, see Methods) across cancer-specific subspaces ranging from 1 to 50 dimensions (Fig. 2e), indicating that most biological signals were preserved during the *DeBias* bias removal process.

To explore the biological relevance of the biased subspaces, we identified the top 25% of features whose variance in healthy individuals was most affected by bias removal, designated as the features most impacted by *DeBias*. The nearest genes of these features were significantly enriched in transcriptional regulatory functions (Additional file 2: Fig. S7), consistent with prior findings that population-specific expression quantitative trait loci (eQTLs) and methylation quantitative trait loci (meQTLs) are enriched in transcriptional regulators and regulatory elements [28–30]. Although these genes overlapped with cancer-related pathways such as Apoptosis, Myc Targets V1, Myc Targets V2, and p53, none of these enrichments reached statistical significance (all Benjamini–Hochberg adjusted Fisher’s exact test *p*-value > 0.15). We also curated race-specific meQTLs for European Americans and African Americans from recent studies [31, 32]. Despite being identified from different experimental platforms, these meQTLs showed slight but non-significant enrichment among the most impacted features (Additional file 1: Table S3). Collectively, these results suggest that the biased subspaces identified by *DeBias* capture biologically meaningful population differences without being strongly linked to cancer-related signals.

We then retrained an LSVM classifier on the adjusted data (see Methods). The AUROC consistently increased across folds for the African American group (0.8891 [0.7835,0.9947], 0.8525 [0.6613,1.0000], and 0.8431 [0.7039,0.9822] in Folds 1–3 and 0.8661 [0.7865,0.9456] overall, with DeLong’s one-sided *p*-value = 0.1369, 0.1063, 0.1547, and 0.0213 against the performance before adjustment; Fig. 2f and Additional file 1: Table S2), indicating enhanced model performance in this underrepresented group. *DeBias* also outperformed other bias-correction approaches, including SMOTE [15], ComBat [17], AE, and adversarial learning [24], in AUROC improvement (Fig. 2f and Additional file 1: Table S2; see Methods). These findings demonstrate that *DeBias* effectively disentangles demographic biases from cancer-associated methylation signals, highlighting its robustness in mitigating racial bias while preserving detection power.

### Validation of *DeBias* performance in independent cohorts

**Fig. 3:**
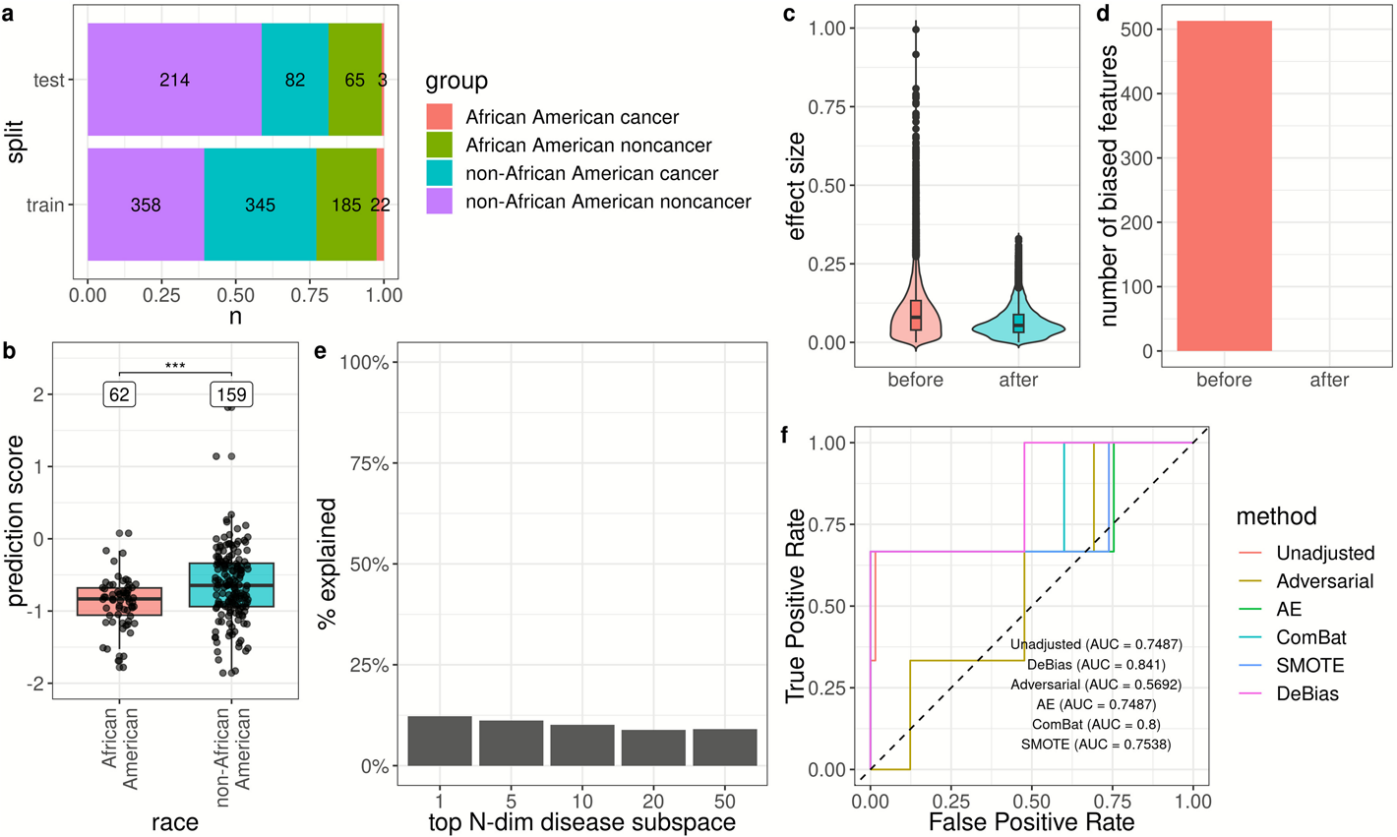
*DeBias* removed racial bias towards underrepresented African American populations in multi-cancer detection on the independent testing set. **a** Composition of the training and testing datasets. **b** Distribution of cancer prediction scores for healthy African American and non-African American individuals in the independent testing set before bias correction. Statistical significance (*p*-value) was assessed using one-sided permutation t-test, comparing cancer prediction scores between African American and non-African American healthy samples. “***” denotes *p* < 0.001. **c** Distribution of race-associated effect size in individual features before and after *DeBias*. The race-associated effect size was evaluated between the African American and non-African American healthy samples. **d** Number of race-associated biased features before and after *DeBias*. **e** Proportion of variance in the top N-dimensional disease subspaces explained by the biased subspaces. **f** ROC curve for African American testing samples before bias correction, after *DeBias* adjustment, and after alternative bias-correction methods

We further validated *DeBias* by training on the original cohort and evaluating performance on an independent *MethylScan* testing set. These samples were collected from independent sources distinct from the original cohort. After independent biospecimen collection and preprocessing, library construction was performed in the same laboratory with the same protocol. This test set included 68 African American (4.4% cancer) and 296 non-African American (22.0% cancer) patients (Fig. 3a; Methods and Additional file 1: Table S4). The noncancer subgroup added complexity, with 18.6% harboring lung benign nodules and 6.5% exhibiting liver diseases. When applying the model trained on the original racially imbalanced data, healthy African Americans received significantly lower cancer prediction scores than healthy non-African Americans (permutation t-test *p*-value = 0.0002, Fig. 3b). Cancer detection AUROC before bias correction was lower for African Americans (0.7487 [0.2697,1.0000]) compared to non-African Americans (0.8547 [0.8032,0.9062]) and the combined cohort (0.8397 [0.7840,0.8954]) on the independent testing set (Fig. 3c and Additional file 1: Table S5).

Using *DeBias*, we identified and removed a 9-dimensional bias subspace from the training data (Additional file 2: Figs. S8 and S9). After *DeBias* correction, race-associated effect sizes across individual features were significantly reduced (permutation t-test *p*-value < 5e-05, Fig. 3c) and the number of biased features decreased to zero (Fig. 3d), confirming the effectiveness of *DeBias* in eliminating feature-level bias. The biased subspaces accounted for 10.3% of the variance across cancer-specific subspaces ranging from 1 to 50 dimensions (Fig. 3e). Furthermore, the most affected features were significantly enriched for transcriptional regulatory functions (Additional file 2: Fig. S10), whereas no cancer-related pathways showed significant enrichment. Collectively, these findings were consistent with the results from the cross-validation analysis, confirming the reproducibility of *DeBias*’ bias-mitigation effects.

*DeBias* improved AUROC for African Americans to 0.8410 [0.5268,1.0000] (with DeLong’s one-sided *p*-value = 0.1454 against the performance before adjustment), surpassing all alternative methods (Fig. 3f and Additional file 1: Table S5). While the improved performance in the minority population was not statistically significant, likely due to the limited number of cancer samples, the consistent gains across validation cohorts underscore *DeBias*’ robustness in mitigating racial bias while maintaining model accuracy.

We also evaluated the performance of *DeBias*-adjusted models in the non–African American population (Additional file 1: Table S2). As expected, applying bias mitigation optimized for the African American population resulted in a modest performance decrease in the non–African American group during cross-validation, likely reflecting a trade-off between bias correction and retention of disease-specific signals. Nevertheless, this decrease was not observed in the independent testing set (Additional file 1: Table S5). Overall, the adjusted model demonstrated comparable performance to the original model in the majority populations.

### Reducing ethnicity-related bias in liver cancer early detection among Hispanic patients

We evaluated *DeBias* in the second clinical scenario: early detection of liver cancer among noncancerous high-risk liver disease patients (e.g. cirrhosis, viral hepatitis, alcohol-related liver disease, and nonalcoholic fatty liver disease). Early detection of liver cancer is critical for improving survival outcomes [33], especially for Hispanic populations, which remain underrepresented in clinical cohorts [34]. Notably, Hispanic individuals experience approximately twice the incidence and rising mortality rates of liver cancer compared with non-Hispanic groups, driven in part by systemic delays in diagnosis [35]. These disparities highlight the urgent need to address ethnicity-related biases in liver cancer detection models.

**Fig. 4:**
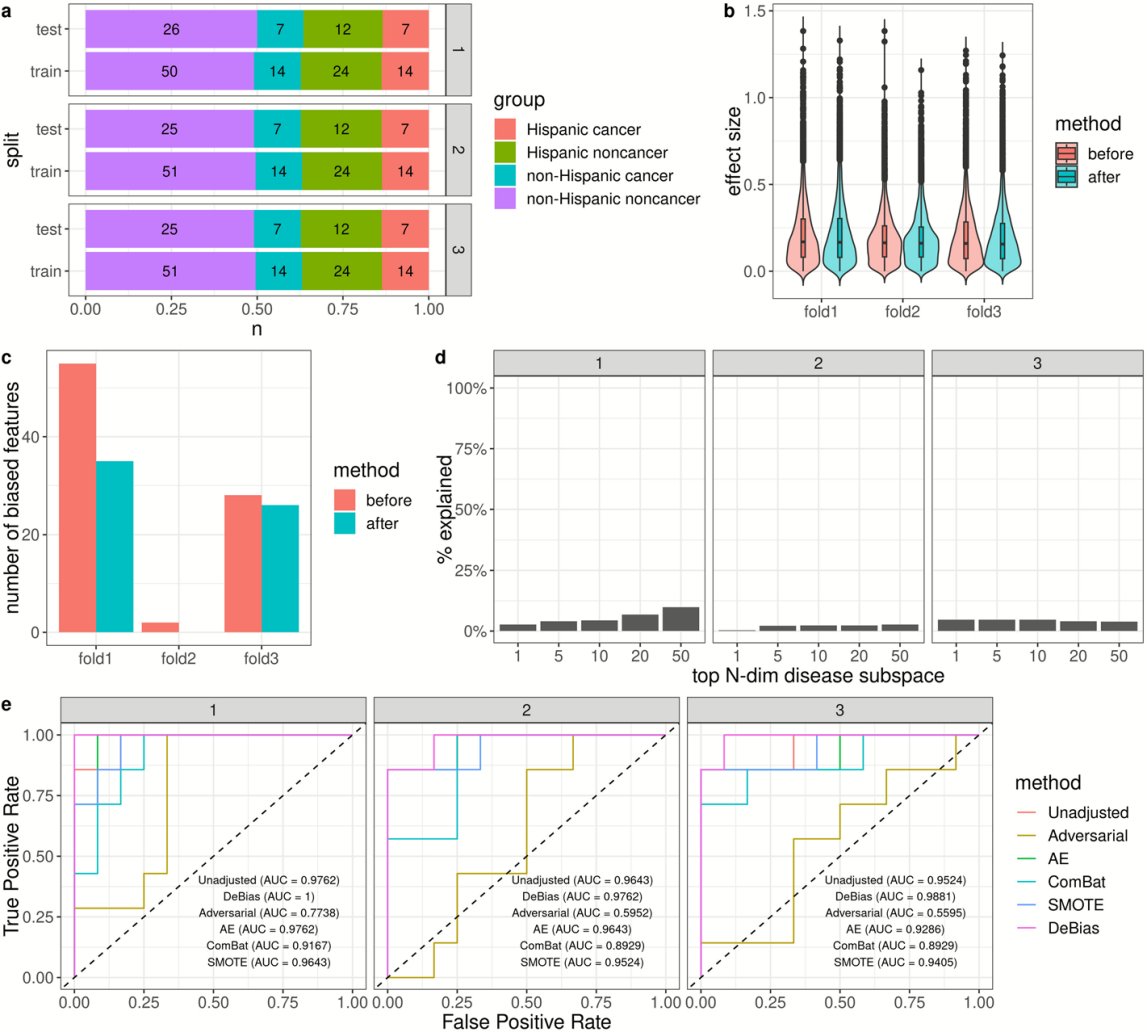
*DeBias* reduces ethnicity-related bias in liver cancer early detection among high-risk noncancerous patients. **a** Composition of the training and testing datasets in the three-fold cross-validation. **b** Distribution of ethnicity-related effect size in individual features before and after *DeBias*. The ethnicity-related effect size was evaluated between the Hispanic and non-Hispanic noncancer samples. **c** Number of ethnicity-related biased features before and after *DeBias*. **d** Proportion of variance in the top N-dimensional disease subspaces explained by the biased subspaces. **e** ROC curve for Hispanic patients before bias correction (Unadjusted), after *DeBias* adjustment, and after alternative bias-correction methods. The facet labels in each subfigure indicated the testing fold ID

We collected a *MethylScan* dataset of 153 plasma samples, including 42 liver cancer cases (52.4% Stage I-II and 47.6% unknown Stage) and 112 noncancerous high-risk controls. This dataset included 97 non-Hispanic (21.6% cancer) and 57 Hispanic (36.8% cancer) patients (Methods and Additional file 1: Table S6). We also performed three-fold cross-validation (Fig. 4a). Because non-Hispanic samples constituted the majority of the training data, the classifier was inherently biased towards optimizing performance for non-Hispanic individuals. As a result, before correction, the cancer detection AUROC was similar across groups, with non-Hispanic patients (0.9341 [0.8426, 1.000], 0.9886 [0.9613,1.0000], and 0.9543 [0.8709,1.0000] in Folds 1–3) and the combined cohort (0.9398 [0.8708,1.0000], 0.9807 [0.9504,1.0000], and 0.9575 [0.9008,1.0000] in Folds 1–3) compared to Hispanic patients (0.9762 [0.9199,1.0000], 0.9643 [0.8853,1.0000], and 0.9524 [0.8509,1.0000] in Folds 1–3) in two of the three folds (Additional file 1: Table S7). The overall performance combining all three folds were comparable across Hispanic (0.9616 [0.9146–1.0000]), non-Hispanic (0.9599 [0.9228–0.9970]), and combined (0.9590 [0.9298–0.9882]).

To address this ethnicity-related bias, we applied *DeBias*, which removed biased subspaces (6, 3, and 3 dimensions for Folds 1–3, respectively) from the liver cancer–specific methylation profiles (Additional file 2: Figs. S11-S16). *DeBias* did not significantly alter ethnicity-associated effect sizes across individual features (permutation t-test *p*-value = 0.985, 0.255, and 0.207 for Folds 1–3, respectively; Fig. 4b), but it reduced the number of biased features (Fig. 4c), indicating measurable bias correction at the feature level. The biased subspaces explained on average 5.5%, 2.0%, and 4.4% of the variance in the cancer-specific subspaces (Fig. 4d), suggesting controllable loss of disease signal.

To interpret the biological relevance of the biased subspaces, we identified the top 25% of features whose variance in noncancer individuals was most affected by bias removal. The nearest genes to these features were significantly enriched for transcriptional regulatory functions (Additional file 2: Fig. S17), which are known to harbor population-specific eQTLs and meQTLs [28–30], whereas no cancer-related pathways showed significant enrichment. Together, these findings indicate that *DeBias* effectively removed demographic-related biases without substantially diminishing cancer-associated signals.

We then retrained a Hispanic-specific liver cancer detection model using the *DeBias*-adjusted methylation features, which improved the AUROC for Hispanic patients across all folds (1.0000 [1.0000,1.0000], 0.9762 [0.9199,1.0000], and 0.9881 [0.9551,1.0000], with DeLong’s one-sided *p*-value = 0.2035, 0.2398, and 0.1877 compared with pre-adjustment performance in Folds 1–3 respectively) and 0.9762 [0.9447, 1.0000] overall (DeLong’s one-sided *p*-value = 0.0938 compared with pre-adjustment performance; Fig. 4e and Additional file 1: Table S7). Although these improvements were not statistically significant, likely due to the limited sample size, *DeBias* outperformed all alternative bias-correction methods (Fig. 4e and Additional file 1: Table S7). When applied to the non-Hispanic population, the *DeBias*-adjusted models showed a moderate AUROC decrease in Fold 1, likely due to the removal of a higher-dimensional biased subspace, but maintained overall performance comparable to the unadjusted models (Additional file 1: Table S7). Nonetheless, the consistent performance gains in the Hispanic population underscore *DeBias’* effectiveness in mitigating ethnicity-related misclassification, representing a key step toward more equitable liver cancer detection across populations.

## Discussion

Machine learning has advanced biomedical research by enabling the analysis of complex high-throughput data, like DNA methylation, yet its clinical utility is hindered by biases rooted in demographically imbalanced training data. These biases are particularly problematic in high-dimensional biological datasets, where confounding demographic and environmental signals can obscure disease-specific patterns. Existing methods for bias correction often fail to address the systemic, population-level biases inherent to clinical datasets, especially in cancer, where patients also exhibit high biological heterogeneity. To overcome this challenge, we developed *DeBias*, a interpretable method that globally isolates and removes bias-associated subspaces across the entire feature space while preserving disease-specific signals. Our results demonstrate that this approach not only mitigates demographic distortions but also enhances model performance in minority populations.

We applied *DeBias* to cancer detection using cfDNA methylation and evaluated it in two high-stakes clinical scenarios: (1) racial bias removal in multi-cancer detection for African Americans within a general population cohort and (2) ethnicity-related bias removal in liver cancer detection for Hispanic patients within a high-risk liver disease cohort. In both cases, *DeBias* achieved a considerable reduction in the number of methylation features exhibiting significant demographic bias, confirming its ability to disentangle confounding signals. In addition, models trained on bias-adjusted data showed consistent improvements in cancer detection accuracy for minority populations, with performance gains validated in an independent cohort. These results demonstrated that our approach could mitigate biases in the training data, leading to a tangible enhancement of the detection performance, reliability, and fairness toward minority populations.

Our study has some limitations. First, our cross-source testing cohorts included only a modest number of samples from the underrepresented populations. To mitigate this, we aggregated an independent testing set for validation. Nevertheless, future studies with larger cohorts could enable a more comprehensive evaluation and possible optimization of parameters for specific clinical applications. Second, *DeBias* uses linear assumptions to simplify bias representation, which may limit its ability to capture complex or nonlinear structures. These assumptions, however, enhance stability and interpretability for the small sample size, where advanced models such as adversarial learning often fail to generalize. As larger, higher-quality datasets become available, nonlinear or multi-factor extensions of *DeBias* could better capture subtle demographic effects. Third, demographic factors such as race and ethnicity were treated as categorical variables, simplifying the biological complexity of ancestry. For instance, genomic admixture (e.g., partial African and European ancestry) may introduce nuanced biases that current population-level corrections cannot fully resolve. Emerging genomic and epigenomic datasets could enable bias correction at higher genomic resolution in the future. Finally, *DeBias* is specifically designed to remove well-defined biases (e.g. demographic biases) and cannot model unknown sources of variation. Methods such as PEER [36], which infer latent factors using Bayesian modeling, are better suited for capturing heterogeneous and unobserved confounders within a dataset. However, unlike *DeBias*, these inferred factors are typically dataset-specific and not readily transferable across cohorts. This distinction reflects that *DeBias* is optimized for targeted, interpretable bias correction rather than for modeling broad latent variability.

We note that although *DeBias* was validated on DNA methylation data for cancer detection, its core principles are applicable to other high-throughput biomedical data types, such as imaging or proteomics, where unexplained demographic biases persist [37]. Extending its application to these domains will require collaborative efforts to benchmark its performance across diverse datasets.

## Conclusions

The *DeBias* framework provides a generalizable approach for mitigating demographic bias in high-dimensional biomedical datasets by identifying and removing bias-associated subspaces while preserving disease-relevant signals. Applied to cfDNA methylation data for cancer detection, *DeBias* reduces feature-level demographic bias and improves model performance for underrepresented populations. In summary, these results support *DeBias* as a step toward developing machine learning models that are more accurate, robust, and equitable for underrepresented populations, thereby enhancing the fairness and generalizability of clinical predictions in real-world healthcare settings.

## Methods

### Data collection

We collected the *MethylScan* data of the plasma samples from 603 noncancer individuals and 439 cancer patients (89 liver, 142 lung, 106 ovary, and 102 gastrointestinal (GI) tract) with sufficient demographic information under the accession code *EGAS00001008125* in the European Genome-Phenome Archive (EGA) [4]. These plasma data were used for the training and cross-source validation of our bias removal approach. We also collected the *MethylScan* data of the matched tumor and normal tissue pairs from 179 cancer patients (80 liver, 60 lung, 13 ovarian, and 26 GI-tract cancer patients) under the accession code *EGAS00001008125* in the EGA [4]. These cancer patients were independent of the cancer patients who contributed their plasma samples. The tissue pair data were used in the cancer marker selection for cancer detection. In addition to these data, we also generated *MethylScan* data of the plasma samples from 248 noncancer individuals and 27 cancer patients (17 liver and 10 GI-tract cancers).

### *MethylScan* library construction and sequencing

*MethylScan* is an improvement of our previously published *cfMethyl-Seq* assay [4]. Briefly, 10 ng from each cfDNA sample was used as the input material. For gDNA samples, 1.5-2ug each was first sonicated by Covaris R230 system (Woburn, MA). Next, size-selection with 0.8x-1.5 × two-step Ampure XP beads was performed to enrich the 150–200 bp fragments. 200 ng of the size-selected gDNA was used as input material. The pre-capture library construction was performed with NEB UltraII DNA library prep kit for Illumina (Ipswich, MA) and the adapter was the methylated EM-seq adapter. Before the first PCR amplification, the gDNA samples were subjected to bisulfite conversion by QIAGEN Epitect kit, and cfDNA samples were subjected to enzymatic conversion by NEBNext enzymatic methyl-seq kit if the hybrid capture panel designed for converted DNA was used downstream. Twist panel hybrid capture was performed with their standard protocol (Twist Bioscience, South San Francisco, CA). The concentration of the post-capture library after the second PCR amplification was measured by Qubit 1xdsDNA HS kit (ThermoFisher, Waltham, MA). Their quality was examined by TBE-UREA PAGE and Bioanalyzer before sequencing. Libraries were then sequenced with 150 bp paired-end reads on Illumina machines by Genewiz, Inc. (South Plainfield, NJ, USA).

### Data preprocessing

The *MethylScan* data of the tissue samples and the plasma samples were preprocessed using the same procedure [4]. We trimmed the Illumina adapters from the raw sequencing reads using Trim-galore [38] using its default settings. Then we performed sequence alignment, deduplication, and methylation calling. We first used Bismark [39] to align the trimmed reads to the reference genome hg19 (GRCh37 (GCA 000001405.1)). Then Bismark was used to remove PCR duplicates based on the mapping locations of the paired-end reads. Bismark methylation extractor [39] was then used to call methylation in the mapped, deduplicated reads. The mapping locations of the read pair, i.e. R1 and R2, were merged to form one fragment.

### Extraction of methylation profiles with the fragment-based signals from the *MethylScan* data

The *MethylScan* assay combined methylation-sensitive restriction enzymes and hybrid capture to selectively deplete WBC-derived cfDNA and enrich tumor-associated cfDNA [4]. Its hybrid capture panel included 153,729 targeted regions and 300 control regions. The target regions were uniformly hypomethylated in WBCs and healthy plasma. Since DNA fragments with unmethylated cutting sites will be digested by the methylation-sensitive restriction enzyme, hypermethylated DNA fragments from non-WBC origins, such as tumor cells, are preferentially preserved and captured [4]. The control regions have no restriction enzyme cutting sites, and thus its coverage was used to normalize the fragment counts in the target regions [4].

For a target region, we extracted hypermethylated DNA fragments that satisfied the following criteria: (1) the DNA fragment overlapped with at least 30% of a target region in the *MethylScan* assay, (2) all restriction enzyme cutting sites on the DNA fragment are hypermethylated, and (3) the averaged methylation level across the CpG sites on the fragment is at least 0.8 (i.e. $\alpha$-value $\ge 0.8$). Then we computed the normalized hypermethylated fragment count in this target region as $\frac{\text{Number of the hypermethylated reads in the target region}}{\text{Average number of captured reads in all control regions}}$. The normalized hypermethylated fragment counts across all target regions were utilized as the full methylation profile of a *MethylScan* sample (either plasma or tissue).

### Detection of cancer markers from matched tumor-normal tissue pairs

In this study, we analyzed 179 matched tumor-normal tissue pairs (80 liver, 60 lung, 13 ovarian, and 26 gastrointestinal tissue pairs) to identify cancer markers, following the marker selection procedure described in [4]. Briefly, we calculated the difference in normalized hypermethylated fragment counts for each tumor-normal pair at each target region. For each cancer type, we ranked all target regions by the average difference across the tissue pairs of that cancer type in descending order and selected the top 5,000 regions with the highest average differences as the cancer-type-specific markers. Then all cancer-type-specific markers were combined together as the pancancer markers. After marker selection, the full methylation profile from a *MethylScan* sample will be subset to the marker regions as its methylation feature profile for cancer detection analysis.

### Bias removal in the methylation feature profiles

Using *DeBias*, we computationally removed methylation subspaces exhibiting demographic bias toward minority populations while preserving those carrying informative disease-related signals. Biased subspaces were identified exclusively from the training data and subsequently removed from the entire dataset, including both training and testing samples. To identify these biased subspaces, *DeBias* employed Cohen’s *d* effect size to quantify demographic differences between control samples from two population groups. When multiple minority populations were present, Cohen’s *f* effect size was used instead. The same approach was applied to quantify disease-associated signals when multiple disease conditions existed. The resulting bias-adjusted feature matrix was then used for model training and evaluation. Because bias identification relied solely on the training data, this procedure effectively prevented data leakage. Given the limited sample size, we retained the default *DeBias* parameters (${es}_{bias}$= 0.1, ${es}_{disease}$= 0.5, and $e{s}_{bias}^{\prime}$= 0.1) for all analyses, and removed subspaces meeting these criteria during the bias correction process. The default parameters were chosen to exclude even small bias effects while preserving medium-to-large disease effects [40, 41]. Our analyses showed that the performance improvement in the minority population after *DeBias* was generally robust under locally varied thresholds of effect sizes (Additional file 2: Fig. S18).

### Identification of biased features

Given a feature matrix, we identified biased features by comparing the control samples in the minority population and majority population with the permutation t-test. Features with a Benjamini–Hochberg adjusted *p*-value of < 0.05 were regarded as biased features.

### Quantification of disease signals in the biased subspaces

To quantify the impact of *DeBias* on disease-related signals, we iteratively identified the top linear discriminants separating case and control samples in the training set using LDA. In each iteration, we identified a linear discriminant, representing the strongest one-dimensional subspace that maximally separates case and control samples. We then orthogonalized this discriminant with respect to previously identified discriminants, projected the data onto this discriminant, removed the projection, and used the residual data to identify the next linear discriminant. Repeating this procedure allowed us to greedily construct a disease-specific subspace of N dimensions with the highest disease classification power.

Next, we assessed how much of the disease subspace could be explained by the biased subspace identified by *DeBias*. To do this, we performed a redundancy analysis (RDA) using the R package vegan and calculated the adjusted ${R}^{2}$, representing the proportion of variance in the disease subspace explained by the biased subspace. This value was interpreted as the loss of disease-related signal attributable to the bias removal procedure.

### Enrichment analyses of the biased subspaces

To investigate the biological relevance of the biased subspaces, we first identified the features—i.e., the average methylation levels across specific genomic regions—that were most impacted by bias removal. Specifically, we calculated the percentage of variance change for each feature before and after applying *DeBias*. The top 25% of features with the largest variance change were considered the most affected features.

Next, we mapped the genomic regions corresponding to these most affected features to the nearest gene transcription start site (TSS) using gene annotations from the R package TxDb.Hsapiens.UCSC.hg19.knownGene. Gene enrichment analysis was then performed against the Gene Ontology Biological Process database and cancer-related pathways from MSigDB (Molecular Signatures Database) using the R package enrichR [42]. Enriched terms and pathways were reported if the Benjamini–Hochberg adjusted *p*-value from Fisher’s exact test was less than 0.001 (Additional file 2: Figs. S7, S10, and S17).

In addition, we curated race-specific meQTLs for European Americans and African Americans from recent studies [31, 32]. We used Fisher’s exact test to assess whether these meQTLs were enriched in the genomic regions corresponding to the most affected features, compared with the full set of cancer detection markers.

### Building a cancer detection model for individuals of the minority population

We trained an LSVM classifier with an L2 penalty on the training plasma samples using the identified cancer markers [4]. The regularization parameter was set to $C=1$, and all other hyperparameters followed the default settings of the Python scikit-learn machine learning package [43]. Before classification, all training and testing data were log-transformed using the function $\mathrm{log}\left(1+1000\cdot \mathrm{X}\right)$ [4].

For the multi-cancer detection task, we trained an LSVM model on the pan-cancer markers. The trained LSVM model output the probability of a testing sample having cancer. For the liver cancer detection task, we trained an LSVM model using the liver-cancer-specific markers. The trained LSVM model output the probability of a testing sample having liver cancer. Note that we used the identical processing steps on the original and bias-adjusted feature matrices to train and evaluate the classification models.

### Comparison with existing methods

To compare *DeBias* against existing bias correction methods, we applied SMOTE [15], ComBat [17], AE, and adversarial learning [24] to the same cancer detection tasks.

ComBat was designed to remove technical biases (e.g. batch effects) during data preprocessing. We estimated the bias correction parameters using the training set and applied these parameters to both the training and testing sets. The adjusted training data were then used to train a linear support vector machine (LSVM) classifier with an L2 penalty ($C=1$), which was applied to the minority population in the testing set.

SMOTE was an oversampling technique that balances the training data by generating synthetic samples to augment the minority group. We applied SMOTE to the training set to generate these synthetic samples, resulting in bias-adjusted training data. As with ComBat, an LSVM classifier with an L2 penalty ($C=1$) was trained on the adjusted data and applied to the minority population in the testing set.

AE and adversarial learning were designed to mitigate demographic biases during model training. The original training data were input into the AE and adversarial models, both of which were constructed using lightweight architectures due to the limited sample size.

The AE model had three components (Additional file 2: Fig. S19): (1) Encoder contained three layers, an input layer followed by two fully connected dense layers with 1024 and 256 nodes, respectively. The encoder processed methylation features and output a 256-dimensional latent representation. (2) Decoder consisted of three layers. The first was a fully connected dense layer with 257 nodes that accepted both the 256-dimensional latent representation and 1-dimensional demographic information as input. The inclusion of demographic information in the decoder encourages the encoder to extract representations that are invariant to demographic characteristics. The subsequent dense layers contained 1024 nodes and a final output layer matching the size of the methylation feature set. (3) Detector included three fully connected layers. The first layer had 256 nodes and received the encoder’s latent representation. The second layer contained 64 nodes, and the third (output) layer consisted of a single node with a sigmoid activation function. All other layers used the rectified linear unit (ReLU) activation. The decoder used mean squared error as its loss function, while the detector used binary cross-entropy. The overall loss function was a weighted sum of the decoder loss (weight = 100) and the detector loss (weight = 1). The model was trained using the Adam optimizer with a learning rate of 0.001, weight decay of 0.0001, batch size of 32, and trained for 30 epochs.

For adversarial learning, we adopted the architecture proposed in [24]. Given the high dimensionality of the methylation features, we increased the number of nodes in the hidden layer of the predictor to 1000, while keeping all other parameters at their default settings.

Both the AE and adversarial learning models output the predicted probability of a testing sample having cancer.

## Supplementary Information

Additional file 1: Tables S1–S7. A compiled multi-tab Excel file containing all supplementary tables referenced in the manuscript, including clinical and demographic characteristics of all datasets, as well as AUROC values and corresponding confidence intervals for all methods across all datasets.

Additional file 2: Figs. S1–S19. A single file containing all supplementary figures referenced in the manuscript. Captions and legends for each supplementary figure are included within the file.

## Data Availability

DeBias is publicly available under the MIT license (OSI Approved License) at Github: https://github.com/DashuoLI/debias [44]. The code is also archived with a DOI at Zenodo: https://doi.org/10.5281/zenodo.18064971 [45] under the MIT license. The raw sequencing data of the plasma and tissue samples are deposited in the EGA under the accession numbers EGAS00001008125 [4] and EGAS00001008127 [46]. These datasets are available under controlled access; access requests can be submitted through the EGA website.

## References

[1] Li S, Noor ZS, Zeng W, Stackpole ML, Ni X, Zhou Y, et al. Sensitive detection of tumor mutations from blood and its application to immunotherapy prognosis. Nat Commun. 2021;12(1):4172. 10.1038/s41467-021-24457-2. PMID: 34234141; PMCID: PMC8263778. 34234141 10.1038/s41467-021-24457-2 PMC8263778

[2] Zviran A, Schulman RC, Shah M, Hill STK, Deochand S, Khamnei CC, et al. Genome-wide cell-free DNA mutational integration enables ultra-sensitive cancer monitoring. Nat Med. 2020;26(7):1114–24. 10.1038/s41591-020-0915-3. Epub 2020 Jun 1. PMID: 32483360; PMCID: PMC8108131. 32483360 10.1038/s41591-020-0915-3 PMC8108131

[3] Li S, Zeng W, Ni X, Zhou Y, Stackpole ML, Noor ZS, et al. CfTrack: a method of exome-wide mutation analysis of cell-free DNA to simultaneously monitor the full spectrum of cancer treatment outcomes including MRD, recurrence, and evolution. Clin Cancer Res. 2022;28(9):1841–53. 10.1158/1078-0432.CCR-21-1242. PMID: 35149536; PMCID: PMC9126584. 35149536 10.1158/1078-0432.CCR-21-1242 PMC9126584

[4] Zeng W, Liu CC, Li S, Zhou Y, Stackpole ML, Xiao Y, et al. Toward the simultaneous detection of multiple diseases with a highly cost-effective cell-free DNA methylome test. in press at PNAS, doi. 10.1073/pnas.2518347123. 10.1073/pnas.2518347123 PMC13080018 41941615

[5] Gao Q, Lin YP, Li BS, Wang GQ, Dong LQ, Shen BY, et al. Unintrusive multi-cancer detection by circulating cell-free DNA methylation sequencing (THUNDER): development and independent validation studies. Ann Oncol. 2023;34(5):486–95. 10.1016/j.annonc.2023.02.010. Epub 2023 Feb 26. PMID: 36849097. 36849097 10.1016/j.annonc.2023.02.010

[6] Cristiano S, Leal A, Phallen J, Fiksel J, Adleff V, Bruhm DC, et al. Genome-wide cell-free DNA fragmentation in patients with cancer. Nature. 2019;570(7761):385–9. 10.1038/s41586-019-1272-6. Epub 2019 May 29. PMID: 31142840; PMCID: PMC6774252. 31142840 10.1038/s41586-019-1272-6 PMC6774252

[7] Shen SY, Singhania R, Fehringer G, Chakravarthy A, Roehrl MHA, Chadwick D, et al. Sensitive tumour detection and classification using plasma cell-free DNA methylomes. Nature. 2018;563(7732):579–83. 10.1038/s41586-018-0703-0. Epub 2018 Nov 14. PMID: 30429608. 30429608 10.1038/s41586-018-0703-0

[8] Obermeyer Z, Powers B, Vogeli C, Mullainathan S. Dissecting racial bias in an algorithm used to manage the health of populations. Science. 2019;366(6464):447–53. 10.1126/science.aax2342. PMID: 31649194. 31649194 10.1126/science.aax2342

[9] Dankwa-Mullan I, Weeraratne D. Artificial intelligence and machine learning technologies in cancer care: addressing disparities, bias, and data diversity. Cancer Discov. 2022;12(6):1423–7. 10.1158/2159-8290.CD-22-0373. PMID: 35652218; PMCID: PMC9662931. 35652218 10.1158/2159-8290.CD-22-0373 PMC9662931

[10] Afrose S, Song W, Nemeroff CB, Lu C, Yao DD. Subpopulation-specific machine learning prognosis for underrepresented patients with double prioritized bias correction. Commun Med. 2022;2(1):111. 10.1038/s43856-022-00165-w. PMID: 36059892; PMCID: PMC9436942. 36059892 10.1038/s43856-022-00165-w PMC9436942

[11] Rajkomar A, Hardt M, Howell MD, Corrado G, Chin MH. Ensuring fairness in machine learning to advance health equity. Ann Intern Med. 2018;169(12):866–72. 10.7326/M18-1990. Epub 2018 Dec 4. PMID: 30508424; PMCID: PMC6594166. 30508424 10.7326/M18-1990 PMC6594166

[12] Huang J, Galal G, Etemadi M, Vaidyanathan M. Evaluation and mitigation of racial bias in clinical machine learning models: scoping review. JMIR Medical Informatics. 2022;10(5):e36388. 10.2196/36388. PMID: 35639450; PMCID: PMC9198828. 35639450 10.2196/36388 PMC9198828

[13] Park JI, Bozkurt S, Park JW, Lee S. Evaluation of race/ethnicity-specific survival machine learning models for Hispanic and Black patients with breast cancer. BMJ Health & Care Informatics. 2023;30(1):e100666. 10.1136/bmjhci-2022-100666. PMID: 36653067; PMCID: PMC9853120. 10.1136/bmjhci-2022-100666 PMC9853120 36653067

[14] Van Hulse J, Khoshgoftaar TM, Napolitano A. Experimental perspectives on learning from imbalanced data. In: Proceedings of the 24th international conference on machine learning 2007. p. 935–942.

[15] Chawla NV, Bowyer KW, Hall LO, Kegelmeyer WP. SMOTE: synthetic minority over-sampling technique. J Artif Intell Res. 2002;16:321–57.

[16] Leek JT, Johnson WE, Parker HS, Jaffe AE, Storey JD. The sva package for removing batch effects and other unwanted variation in high-throughput experiments. Bioinformatics. 2012;28(6):882–3. 10.1093/bioinformatics/bts034. Epub 2012 Jan 17. PMID: 22257669; PMCID: PMC3307112. 22257669 10.1093/bioinformatics/bts034 PMC3307112

[17] Johnson WE, Li C, Rabinovic A. Adjusting batch effects in microarray expression data using empirical Bayes methods. Biostatistics. 2007;8(1):118–27. 10.1093/biostatistics/kxj037. Epub 2006 Apr 21. PMID: 16632515. 16632515 10.1093/biostatistics/kxj037

[18] Zhang Y, Parmigiani G, Johnson WE. *ComBat-seq*: batch effect adjustment for RNA-seq count data. NAR Genom Bioinform. 2020;2(3):lqaa078. 10.1093/nargab/lqaa078. Epub 2020 Sep 21. PMID: 33015620; PMCID: PMC7518324. 33015620 10.1093/nargab/lqaa078 PMC7518324

[19] Kapsner LA, Zavgorodnij MG, Majorova SP, Hotz-Wagenblatt A, Kolychev OV, Lebedev IN, et al. Biascorrector: fast and accurate correction of all types of experimental biases in quantitative DNA methylation data derived by different technologies. Int J Cancer. 2021;149(5):1150–65. 10.1002/ijc.33681. Epub 2021 May 26. PMID: 33997972. 33997972 10.1002/ijc.33681

[20] Khor S, Haupt EC, Hahn EE, Lyons LJL, Shankaran V, Bansal A. Racial and ethnic bias in risk prediction models for colorectal cancer recurrence when race and ethnicity are omitted as predictors. JAMA Netw Open. 2023;6(6): e2318495. 10.1001/jamanetworkopen.2023.18495. PMID: 37318804; PMCID: PMC10273018. 37318804 10.1001/jamanetworkopen.2023.18495 PMC10273018

[21] Moyer D, Gao S, Brekelmans R, Galstyan A, Ver Steeg G. Invariant representations without adversarial training. In: Advances in neural information processing systems. 2018. p. 9084–9093.

[22] Creager E, Madras D, Jacobsen JH, Weis M, Swersky K, Pitassi T, Zemel R. Flexibly fair representation learning by disentanglement. In: International conference on machine learning. 2019. p. 1436–1445.

[23] Kingma DP, Welling M. Auto-encoding variational bayes. 2013.

[24] Yang J, Soltan AA, Eyre DW, Yang Y, Clifton DA. An adversarial training framework for mitigating algorithmic biases in clinical machine learning. NPJ Digit Med. 2023;6(1):55. 36991077 10.1038/s41746-023-00805-y PMC10050816

[25] Jolliffe IT, Cadima J. Principal component analysis: a review and recent developments. Philos Trans R Soc Lond A Math Phys Eng Sci. 2016;374(2065):20150202. 10.1098/rsta.2015.0202. PMID: 26953178; PMCID: PMC4792409. 10.1098/rsta.2015.0202 PMC4792409 26953178

[26] Schrag D, Beer TM, McDonnell CH 3rd, Nadauld L, Dilaveri CA, Reid R, et al. Blood-based tests for multicancer early detection (PATHFINDER): a prospective cohort study. Lancet. 2023;402(10409):1251–60. 10.1016/S0140-6736(23)01700-2. PMID: 37805216; PMCID: PMC11027492. 37805216 10.1016/S0140-6736(23)01700-2 PMC11027492

[27] Lennon AM, Buchanan AH, Kinde I, Warren A, Honushefsky A, Cohain AT, et al. Feasibility of blood testing combined with PET-CT to screen for cancer and guide intervention. Science. 2020;369(6499):eabb9601. 10.1126/science.abb9601. Epub 2020 Apr 28. PMID: 32345712; PMCID: PMC7509949. 32345712 10.1126/science.abb9601 PMC7509949

[28] Husquin LT, Rotival M, Fagny M, Quach H, Zidane N, McEwen LM, et al. Exploring the genetic basis of human population differences in DNA methylation and their causal impact on immune gene regulation. Genome Biol. 2018;19(1):222. 10.1186/s13059-018-1601-3. PMID: 30563547; PMCID: PMC6299574. 30563547 10.1186/s13059-018-1601-3 PMC6299574

[29] Koyama S, Liu X, Koike Y, Hikino K, Koido M, Li W, et al. Population-specific putative causal variants shape quantitative traits. Nat Genet. 2024;56(10):2027–35. 10.1038/s41588-024-01913-5. Epub 2024 Oct 3. PMID: 39363016; PMCID: PMC11525193. 39363016 10.1038/s41588-024-01913-5 PMC11525193

[30] Kachuri L, Mak ACY, Hu D, Eng C, Huntsman S, Elhawary JR, et al. Gene expression in African Americans, Puerto Ricans and Mexican Americans reveals ancestry-specific patterns of genetic architecture. Nat Genet. 2023;55(6):952–63. 10.1038/s41588-023-01377-z. Epub 2023 May 25. PMID: 37231098; PMCID: PMC10260401. 37231098 10.1038/s41588-023-01377-z PMC10260401

[31] Min JL, Hemani G, Hannon E, Dekkers KF, Castillo-Fernandez J, Luijk R, et al. Genomic and phenotypic insights from an atlas of genetic effects on DNA methylation. Nat Genet. 2021;53(9):1311–21. 10.1038/s41588-021-00923-x. Epub 2021 Sep 6. PMID: 34493871; PMCID: PMC7612069. 34493871 10.1038/s41588-021-00923-x PMC7612069

[32] Li B, Aouizerat BE, Cheng Y, Anastos K, Justice AC, Zhao H, et al. Incorporating local ancestry improves identification of ancestry-associated methylation signatures and meQTLs in African Americans. Commun Biol. 2022;5(1):401. 10.1038/s42003-022-03353-5. PMID: 35488087; PMCID: PMC9054854. 35488087 10.1038/s42003-022-03353-5 PMC9054854

[33] Pomenti S, Gandle C, Abu Sbeih H, Phipps M, Livanos A, Guo A, et al. Hepatocellular carcinoma in Hispanic patients: trends and outcomes in a large United States cohort. Hepatol Commun. 2020;4(11):1708–16. 10.1002/hep4.1575. PMID: 33163839; PMCID: PMC7603535. 33163839 10.1002/hep4.1575 PMC7603535

[34] Monge C, Greten TF. Underrepresentation of Hispanics in clinical trials for liver cancer in the United States over the past 20 years. Cancer Med. 2024;13(1):e6814. 10.1002/cam4.6814. Epub 2023 Dec 20. PMID: 38124450; PMCID: PMC10807616. 38124450 10.1002/cam4.6814 PMC10807616

[35] Islami F, Miller KD, Siegel RL, Fedewa SA, Ward EM, Jemal A. Disparities in liver cancer occurrence in the United States by race/ethnicity and state. CA Cancer J Clin. 2017;67(4):273–89. 10.3322/caac.21402. Epub 2017 Jun 6. PMID: 28586094. 28586094 10.3322/caac.21402

[36] Stegle O, Parts L, Piipari M, Winn J, Durbin R. Using probabilistic estimation of expression residuals (PEER) to obtain increased power and interpretability of gene expression analyses. Nat Protoc. 2012;7(3):500–7. 10.1038/nprot.2011.457. PMID: 22343431; PMCID: PMC3398141. 22343431 10.1038/nprot.2011.457 PMC3398141

[37] Gichoya JW, Banerjee I, Bhimireddy AR, Burns JL, Celi LA, Chen LC, et al. AI recognition of patient race in medical imaging: a modelling study. Lancet Digit Health. 2022;4(6):e406–14. 10.1016/S2589-7500(22)00063-2. Epub 2022 May 11. PMID: 35568690; PMCID: PMC9650160. 35568690 10.1016/S2589-7500(22)00063-2 PMC9650160

[38] Krueger F. Software “Trim Galore.” http://www.bioinformatics.babraham.ac.uk/projects/trim_galore/.

[39] Krueger F, Andrews SR. Bismark: a flexible aligner and methylation caller for bisulfite-seq applications. Bioinformatics. 2011;27(11):1571–2. 10.1093/bioinformatics/btr167. Epub 2011 Apr 14. PMID: 21493656; PMCID: PMC3102221. 21493656 10.1093/bioinformatics/btr167 PMC3102221

[40] Cohen J. Statistical power analysis for the behavioral sciences, Rev. Lawrence Erlbaum Associates, Inc. 1977.

[41] Sawilowsky SS. New effect size rules of thumb. J Mod Appl Stat Methods. 2009;8(2):26.

[42] Chen EY, Tan CM, Kou Y, Duan Q, Wang Z, Meirelles GV, et al. Enrichr: interactive and collaborative HTML5 gene list enrichment analysis tool. BMC Bioinformatics. 2013;14:128. 10.1186/1471-2105-14-128. PMID: 23586463; PMCID: PMC3637064. 23586463 10.1186/1471-2105-14-128 PMC3637064

[43] Pedregosa F, et al. Scikit-learn: machine learning in Python. J Mach Learn Res. 2011;12:2825–30.

[44] Li S, Zeng W, Li W, Liu CC, Zhou Y, Ni X, et al. Reducing demographic bias in biomedical machine learning for cancer detection using cfDNA methylation. Github; 2025. https://github.com/DashuoLI/debias. 10.1186/s13059-026-04006-0 PMC13104312 41736096

[45] Li S, Zeng W, Li W, Liu CC, Zhou Y, Ni X, et al. Reducing demographic bias in biomedical machine learning for cancer detection using cfDNA methylation. Zenodo. 2025. 10.5281/zenodo.18064971. 10.1186/s13059-026-04006-0 PMC13104312 41736096

[46] Li S, Zeng W, Li W, Liu CC, Zhou Y, Ni X, et al. Reducing demographic bias in biomedical machine learning for cancer detection using cfDNA methylation. Datasets. European Genome-phenome Archive. https://www.ega-archive.org/studies/EGAS00001008127. 2025 10.1186/s13059-026-04006-0 PMC13104312 41736096

