# Supplementary material for: Reducing demographic bias in biomedical machine learning for cancer detection using cfDNA methylation
Source: Genome Biol. 2026 Feb 25;27:141. doi: 10.1186/s13059-026-04006-0 (PMC13104312; doi:10.1186/s13059-026-04006-0)
Supplement: Supplementary file 2 — Additional file 2: Figs. S1–S19. A single file containing all supplementary figures referenced in the manuscript. Captions and legends for each supplementary figure are included within the file. [file 13059_2026_4006_MOESM2_ESM.pdf]

## Supplementary Figures

**Fig S1.** Scatter plots of training samples before and after DeBias correction for cross-validation Fold 1 in the racial bias removal analysis of multi-cancer detection. In the demographic variable, "–1" denotes the minority population and "1" denotes the majority population. In the cancer status variable, "–1" represents noncancer samples and "1" represents cancer samples.

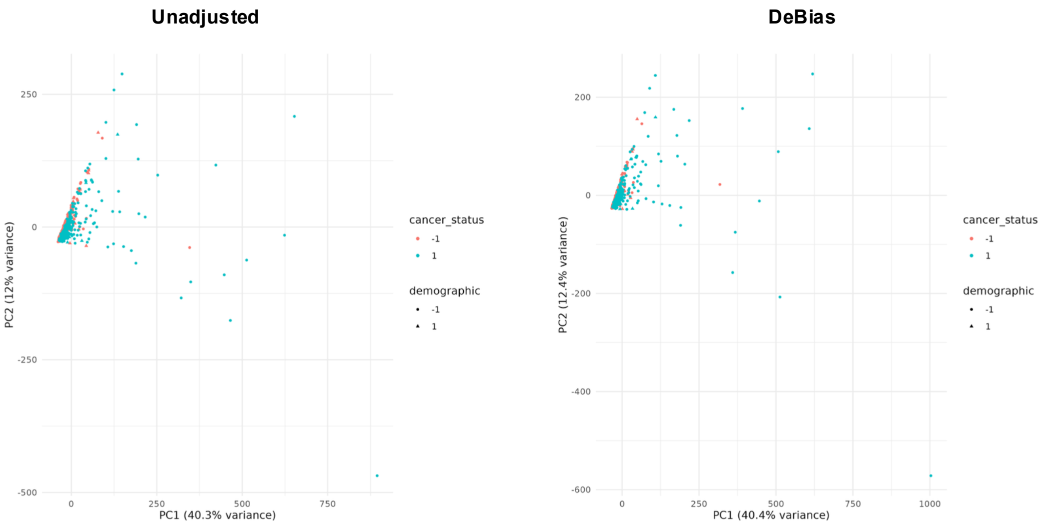

**Fig S2.** Scatter plots of training samples before and after DeBias correction for cross-validation Fold 2 in the racial bias removal analysis of multi-cancer detection. In the demographic variable, "–1" denotes the minority population and "1" denotes the majority population. In the cancer status variable, "–1" represents noncancer samples and "1" represents cancer samples.

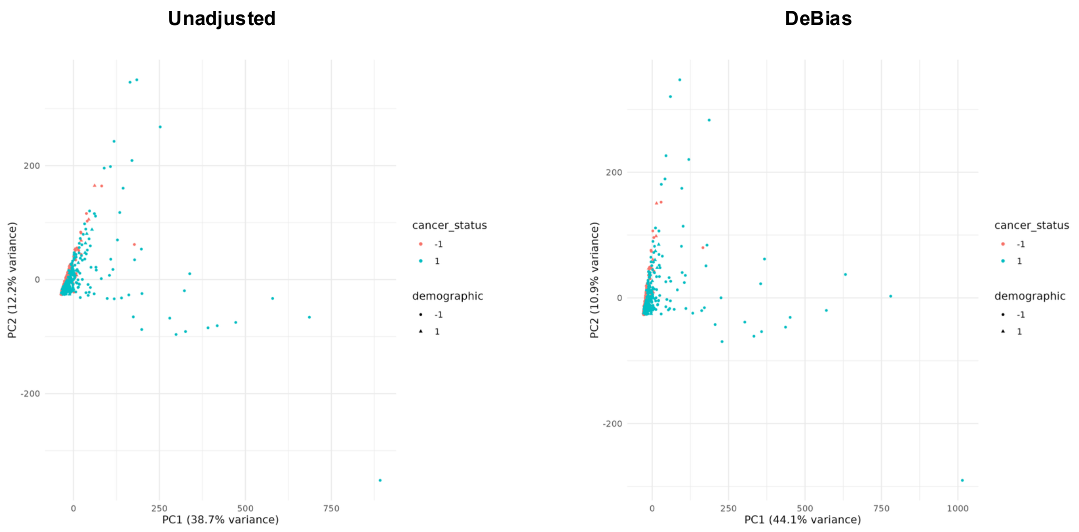

**Fig S3.** Scatter plots of training samples before and after DeBias correction for cross-validation Fold 3 in the racial bias removal analysis of multi-cancer detection. In the demographic variable, "–1" denotes the minority population and "1" denotes the majority population. In the cancer status variable, "–1" represents noncancer samples and "1" represents cancer samples.

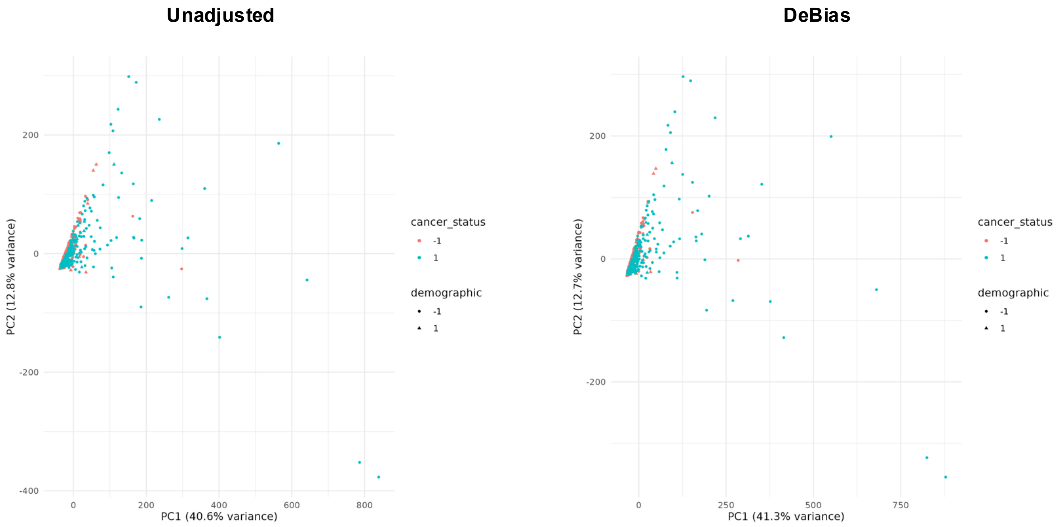

**Fig S4.** Heatmaps of training samples before and after DeBias correction for cross-validation Fold 1 in the racial bias removal analysis of multi-cancer detection.

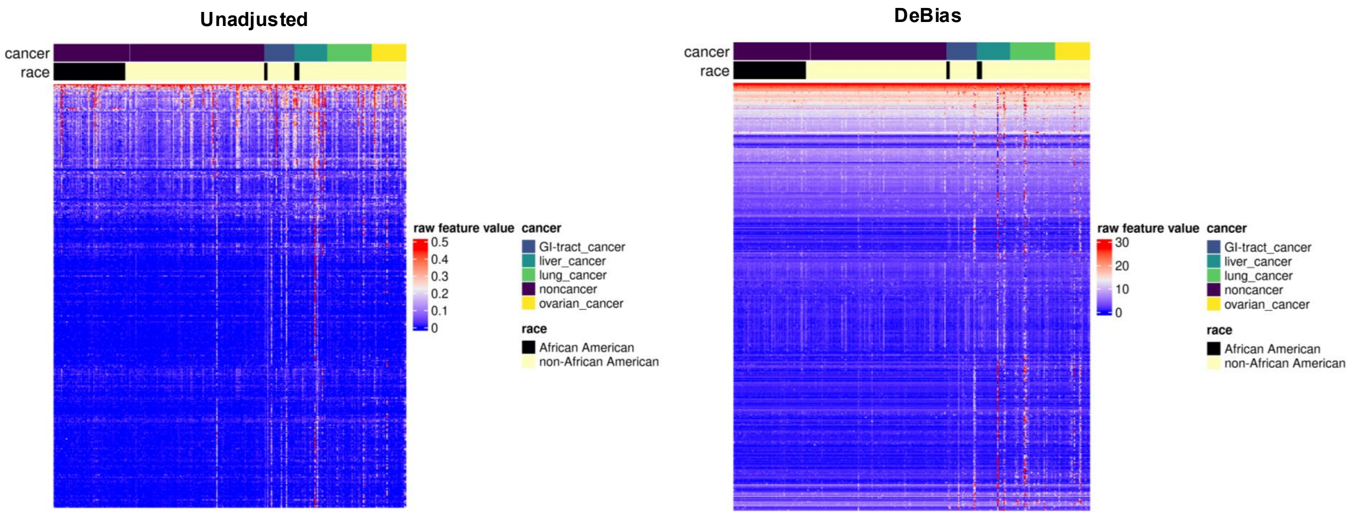

**Fig S5.** Heatmaps of training samples before and after DeBias correction for cross-validation Fold 2 in the racial bias removal analysis of multi-cancer detection.

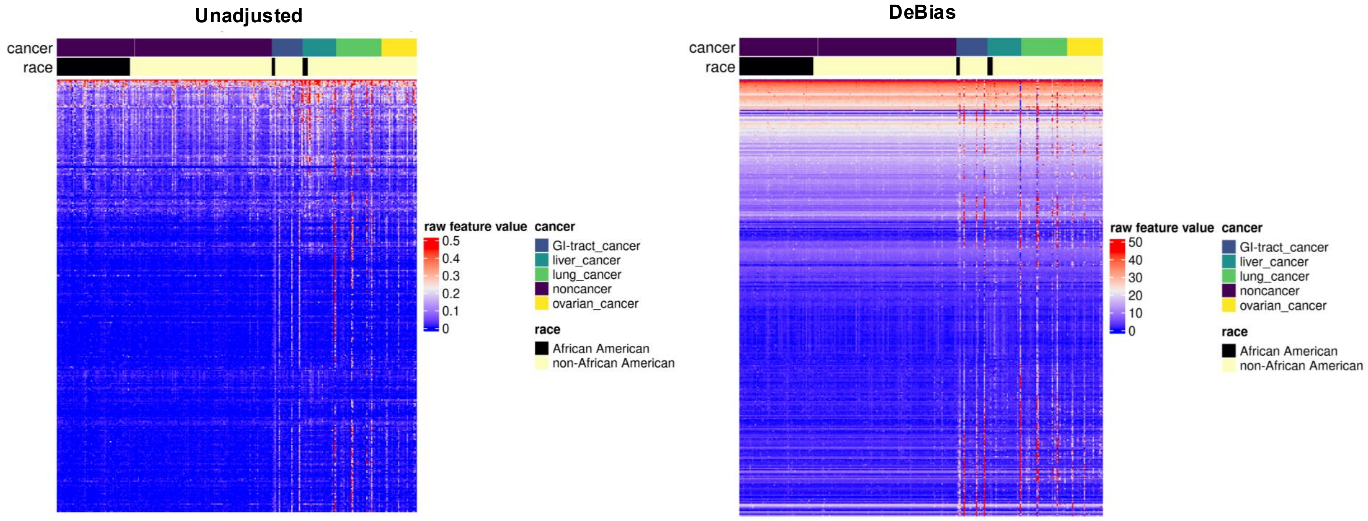

**Fig S6.** Heatmaps of training samples before and after DeBias correction for cross-validation Fold 3 in the racial bias removal analysis of multi-cancer detection.

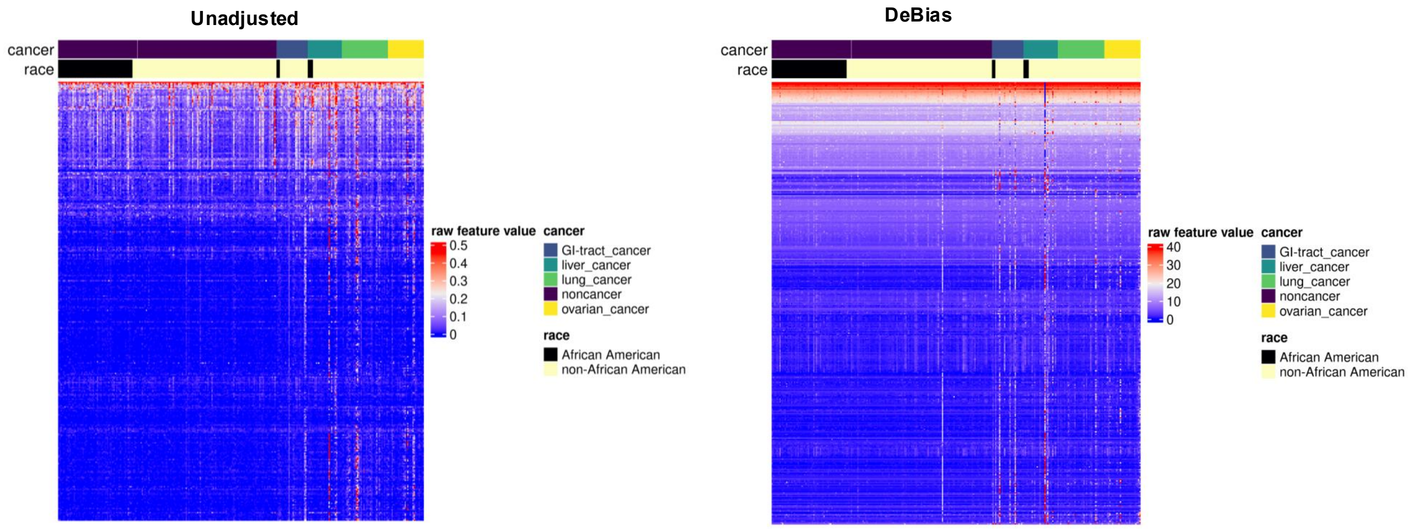

**Fig S7**. Top 15 enriched Gene Ontology (GO) terms (adjusted p-value < 0.001) of the nearest genes corresponding to the most affected features following bias removal across the three cross-validation folds in the multi-cancer detection analysis.

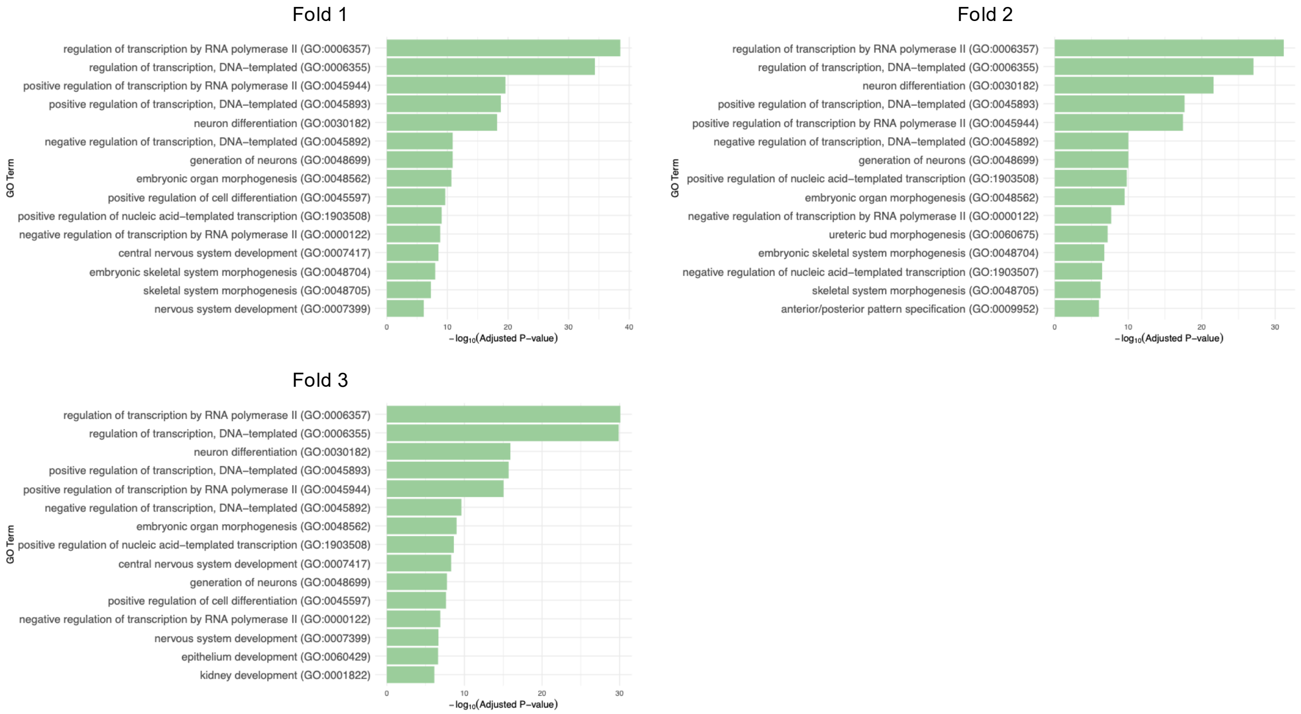

**Fig S8.** Scatter plots of training samples before and after DeBias correction for independent test in the racial bias removal analysis of multi-cancer detection. In the demographic variable, "–1" denotes the minority population and "1" denotes the majority population. In the cancer status variable, "–1" represents noncancer samples and "1" represents cancer samples.

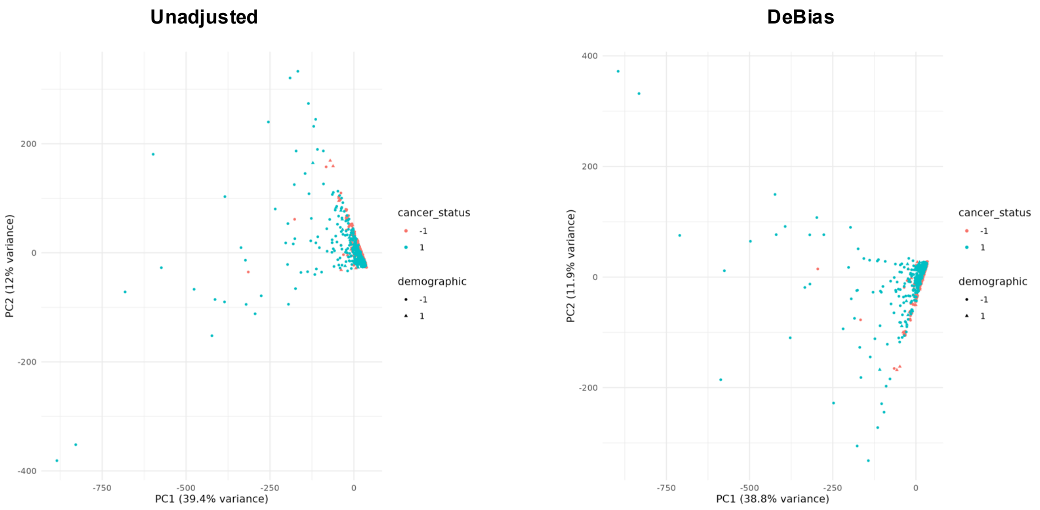

**Fig S9.** Heatmaps of training samples before and after DeBias correction for independent test in the racial bias removal analysis of multi-cancer detection.

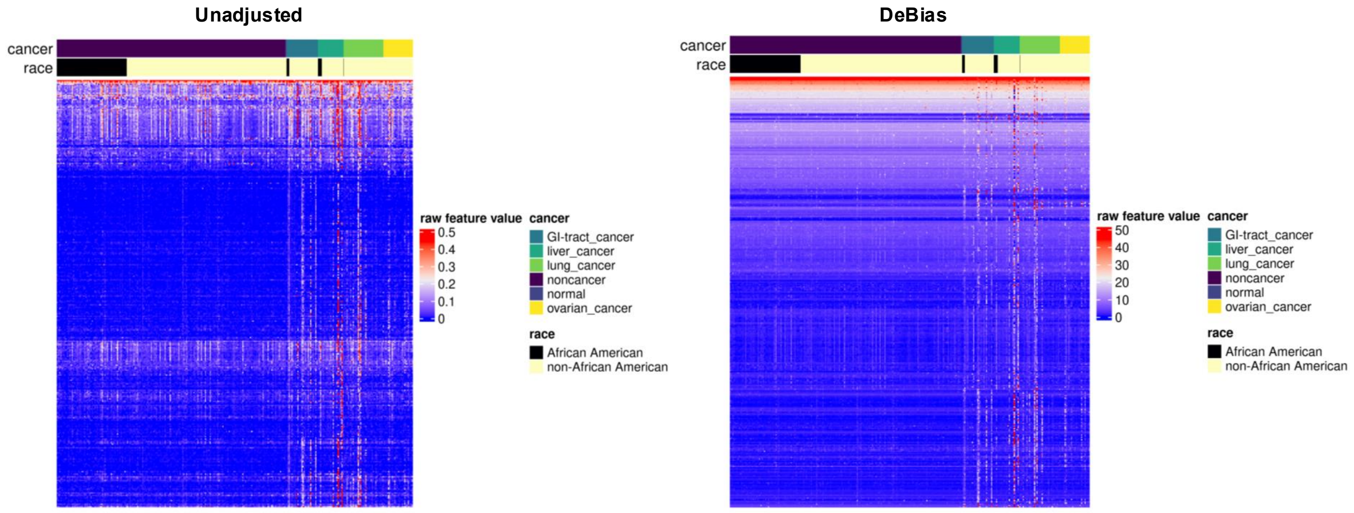

**Fig S10**. Top 15 enriched Gene Ontology (GO) terms (adjusted p-value < 0.001) of the nearest genes corresponding to the most affected features following bias removal across the independent test in the multi-cancer detection analysis.

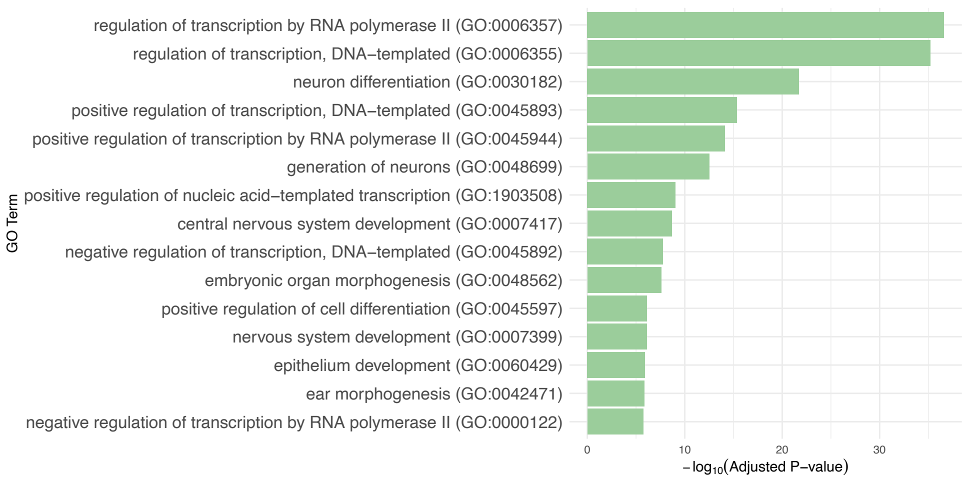

**Fig S11.** Scatter plots of training samples before and after DeBias correction for cross-validation Fold 1 in the ethnicity-related bias removal in liver cancer detection. In the demographic variable, "–1" denotes the minority population and "1" denotes the majority population. In the cancer status variable, "–1" represents noncancer samples and "1" represents cancer samples.

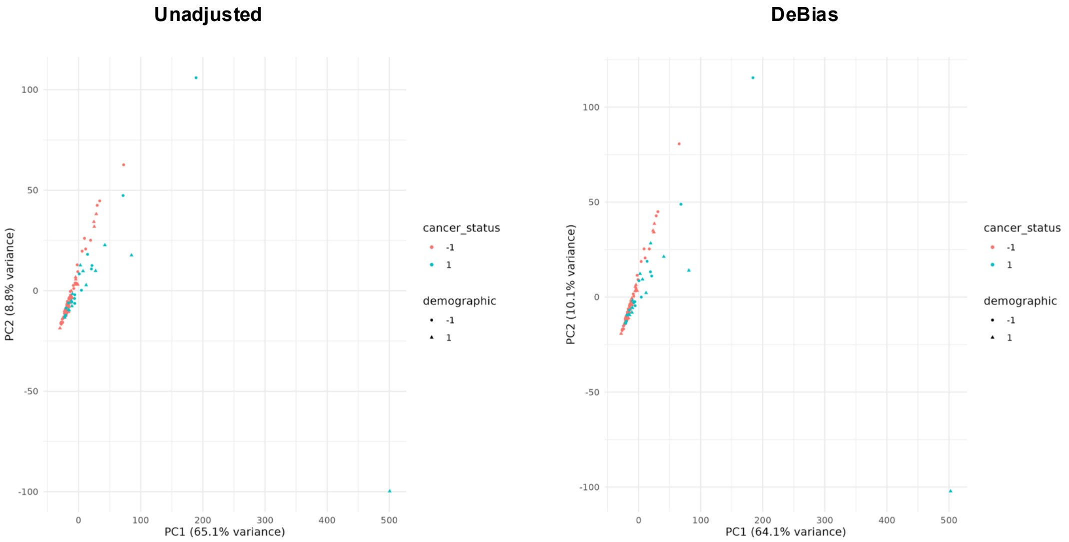

**Fig S12.** Scatter plots of training samples before and after DeBias correction for cross-validation Fold 2 in the ethnicity-related bias removal in liver cancer detection. In the demographic variable, “–1” denotes the minority population and “1” denotes the majority population. In the cancer status variable, “–1” represents noncancer samples and “1” represents cancer samples.

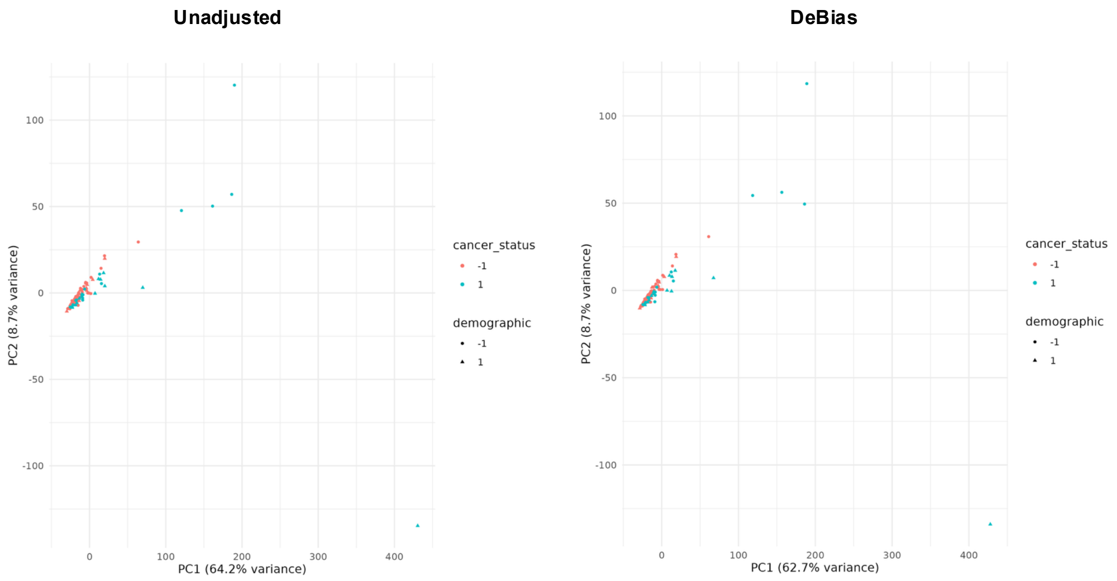

**Fig S13.** Scatter plots of training samples before and after DeBias correction for cross-validation Fold 3 in the ethnicity-related bias removal in liver cancer detection. In the demographic variable, “–1” denotes the minority population and “1” denotes the majority population. In the cancer status variable, “–1” represents noncancer samples and “1” represents cancer samples.

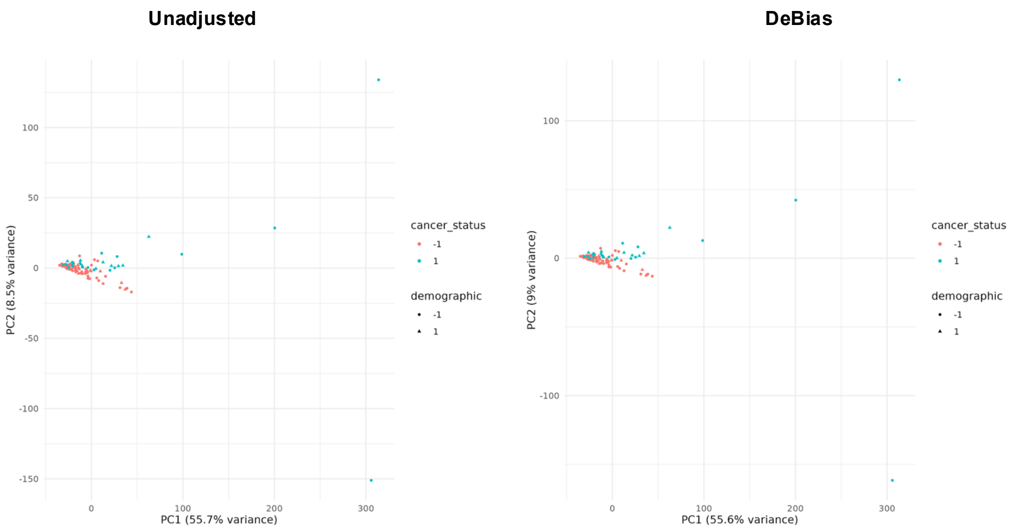

**Fig S14.** Heatmaps of training samples before and after DeBias correction for cross-validation Fold 1 in the ethnicity-related bias removal in liver cancer detection.

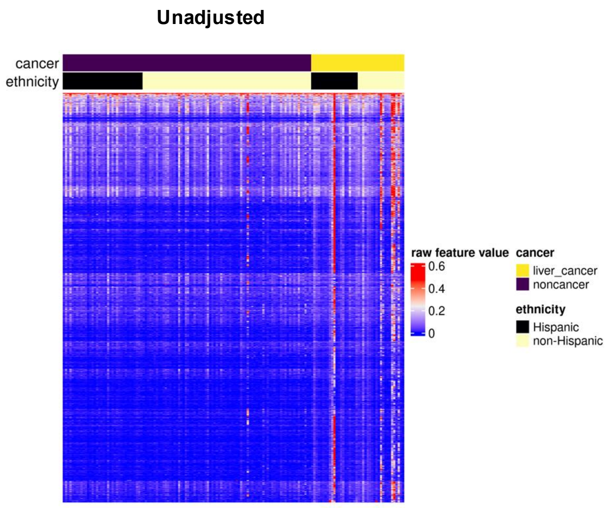

**Fig S15.** Heatmaps of training samples before and after DeBias correction for cross-validation Fold 2 in the ethnicity-related bias removal in liver cancer detection.

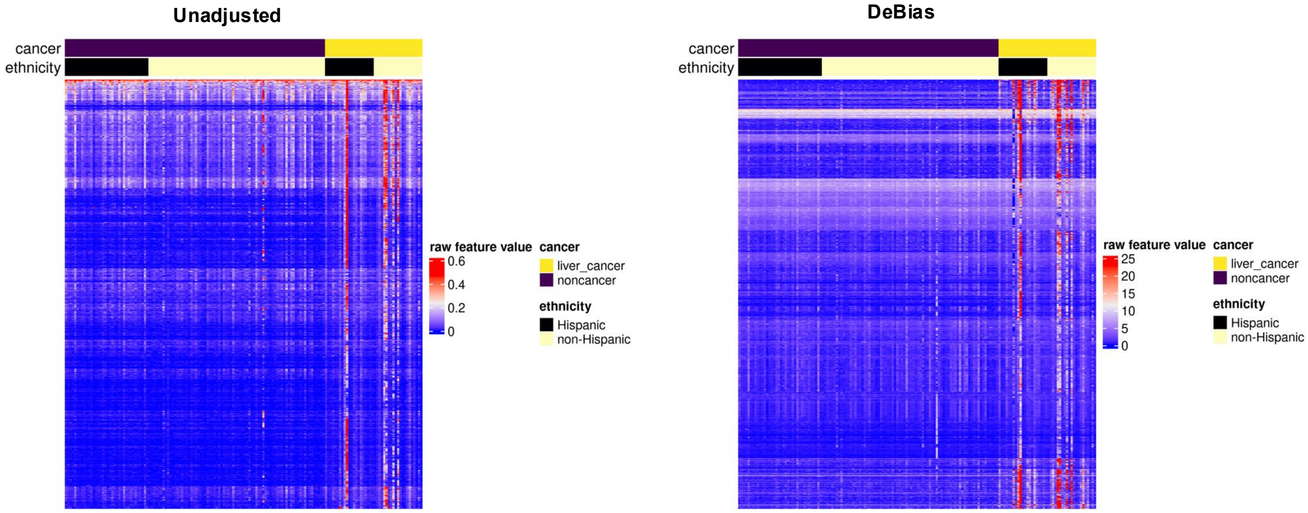

**Fig S16.** Heatmaps of training samples before and after DeBias correction for cross-validation Fold 3 in the ethnicity-related bias removal in liver cancer detection.

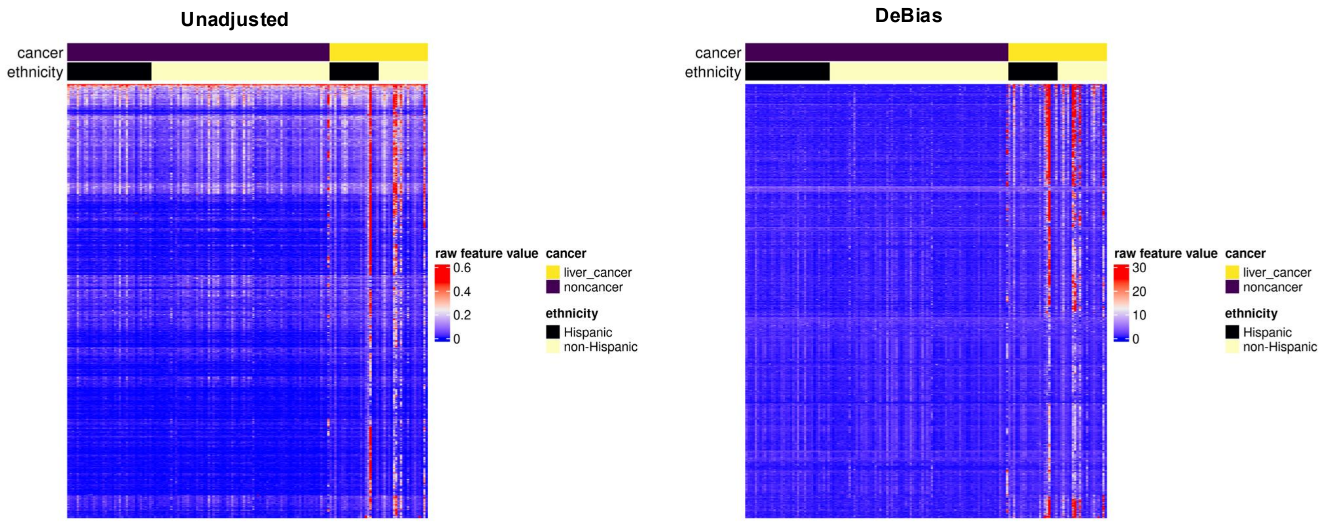

**Fig S17**. Top 15 enriched Gene Ontology (GO) terms (adjusted p-value < 0.001) of the nearest genes corresponding to the most affected features following bias removal across the three cross-validation folds in the liver cancer detection analysis.

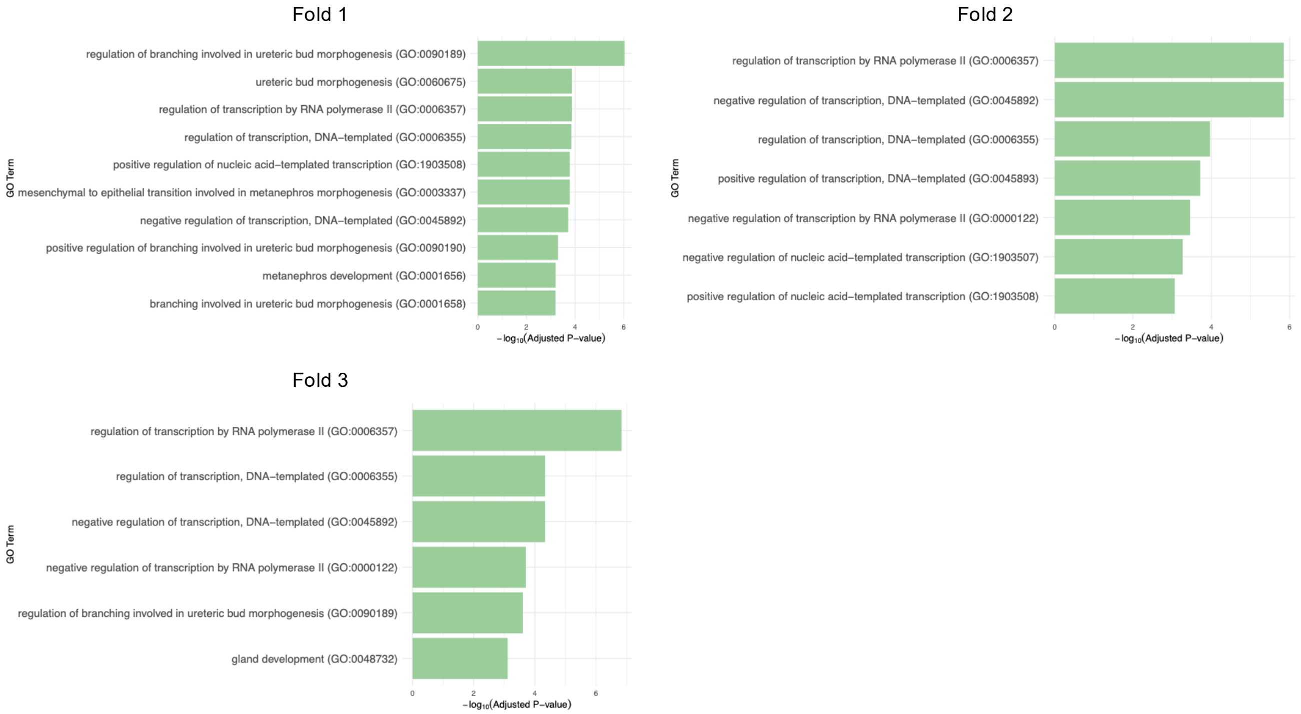

**Fig S18.** Cancer detection AUC in the minority population after DeBias under different threshold settings. The dashed line indicates the performance before bias adjustment.

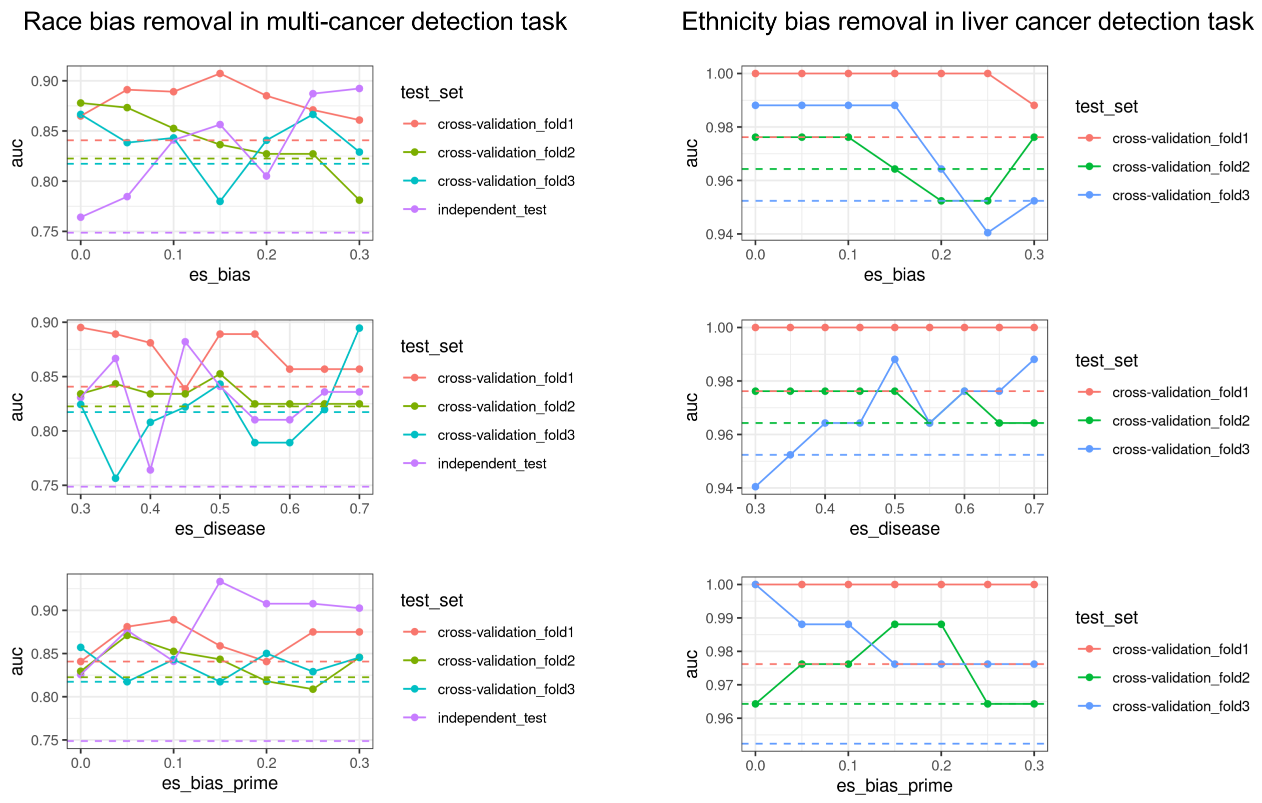

**Fig S19.** Structure Illustration of the variational auto-encoder (VAE).

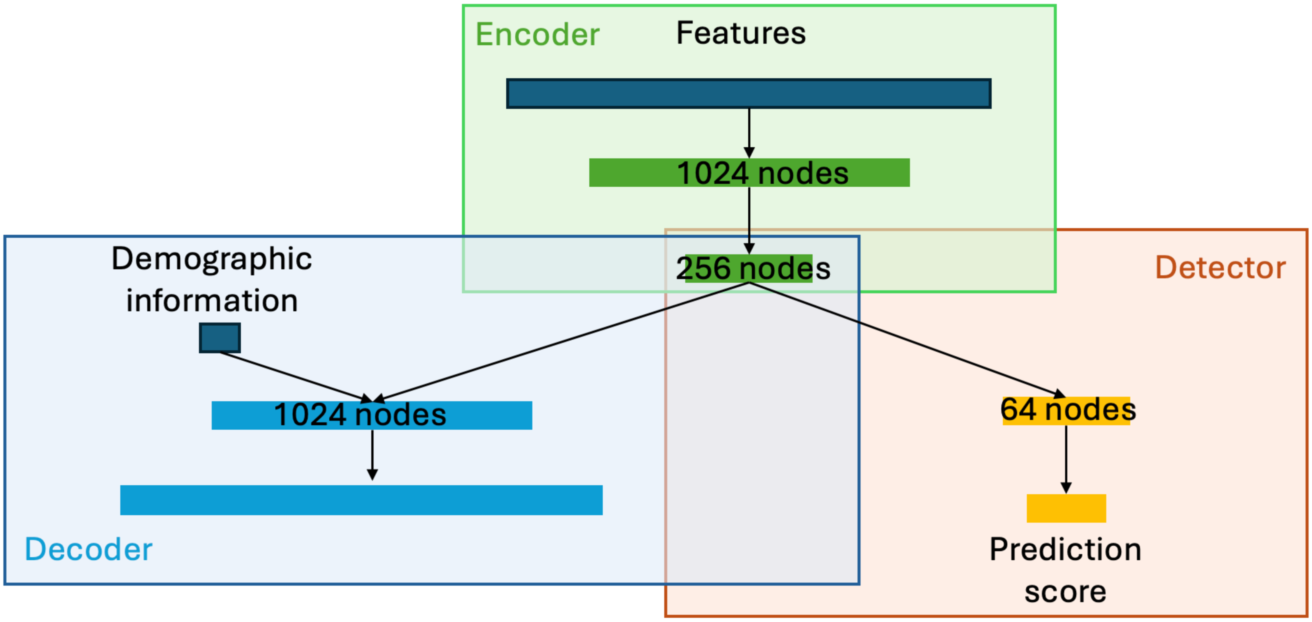


$$\text{Loss} = w_{decoder} * \text{MSE(decoder output, input)} + w_{detector} * \text{BinaryCrossEntropy(detector output, true label)}$$
